# Synthesis and new DNA targeting activity of 6- and 7-*tert*-butylfascaplysins

**DOI:** 10.1038/s41598-024-62358-8

**Published:** 2024-05-23

**Authors:** Sergey A. Dyshlovoy, Wael Y. Mansour, Natalia A. Ramm, Jessica Hauschild, Maxim E. Zhidkov, Malte Kriegs, Alexandra Zielinski, Konstantin Hoffer, Tobias Busenbender, Ksenia A. Glumakova, Pavel V. Spirin, Vladimir S. Prassolov, Derya Tilki, Markus Graefen, Carsten Bokemeyer, Gunhild von Amsberg

**Affiliations:** 1 Laboratory of Experimental Oncology, Department of Oncology, Hematology and Bone Marrow Transplantation With Section Pneumology, Hubertus Wald Tumorzentrum – University Cancer Center Hamburg (UCCH), University Medical Center Hamburg-Eppendorf, Martinistrasse 52, 20246 Hamburg, Germany; 2 Department of Radiotherapy and Radiation Oncology, Hubertus Wald Tumorzentrum – University Cancer Center Hamburg (UCCH), University Medical Center Hamburg-Eppendorf, Martinistrasse 52, 20246 Hamburg, Germany; 3 Mildred Scheel Cancer Career Center HaTriCS4, University Medical Center Hamburg-Eppendorf, Martinistrasse 52, 20246 Hamburg, Germany; 4 Department of Chemistry and Materials, Institute of High Technologies and Advanced Materials, Far Eastern Federal University, FEFU Campus, Ajax Bay 10, 690922 Vladivostok, Russky Island Russian Federation; 5 UCCH Kinomics Core Facility, Hubertus Wald Tumorzentrum – University Cancer Center Hamburg (UCCH), University Medical Center Hamburg-Eppendorf, Martinistrasse 52, 20246 Hamburg, Germany; 6 Department of Cancer Cell Biology, Engelhardt Institute of Molecular Biology, Russian Academy of Sciences, Vavilova 32, 119991 Moscow, Russian Federation; 7 Center for Precision Genome Editing and Genetic Technologies for Biomedicine, Engelhardt Institute of Molecular Biology, Russian Academy of Sciences, Vavilova 32, 119991 Moscow, Russian Federation; 8 Department of Urology, University Medical Center Hamburg-Eppendorf, Martinistrasse 52, 20246 Hamburg, Germany; 9 Department of Urology, Koc University Hospital, 34010 Istanbul, Turkey; 10 Martini-Klinik, Prostate Cancer Center, University Medical Center Hamburg-Eppendorf, Martinistrasse 52, 20246, Hamburg, Germany

**Keywords:** Fascaplysin, Marine compound, Synthesis, Prostate cancer, DNA targeting, Natural products, Synergism, Chemical modification, Prostate cancer, Cancer therapeutic resistance, Chemotherapy, Targeted therapies

## Abstract

Fascaplysin is a red cytotoxic pigment with anticancer properties isolated from the marine sponge *Fascaplysinopsis* sp. Recently, structure–activity relationship analysis reported by our group suggested that selective cytotoxicity of fascaplysin derivatives towards tumor cells negatively correlates with their ability to intercalate into DNA. To validate this hypothesis, we synthesized 6- and 7-*tert*-butylfascaplysins which reveal mitigated DNA-intercalating properties. These derivatives were found to be strongly cytotoxic to drug-resistant human prostate cancer cells, albeit did not demonstrate improved selectivity towards cancer cells when compared to fascaplysin. At the same time, kinome analysis suggested an activation of CHK1/ATR axis in cancer cells shortly after the drug exposure. Further experiments revealed induction of replication stress that is eventually converted to the toxic DNA double-strand breaks, resulting in caspase-independent apoptosis-like cell death. Our observations highlight new DNA-targeting effect of some fascaplysin derivatives and indicate more complex structure–activity relationships within the fascaplysin family, suggesting that cytotoxicity and selectivity of these alkaloids are influenced by multiple factors. Furthermore, combination with clinically-approved inhibitors of ATR/CHK1 as well as testing in tumors particularly sensitive to the DNA damage should be considered in further studies.

## Introduction

Marine invertebrates, and marine sponges in particular, have emerged as a rich source of novel molecules with unique structures and promising biological activities^1,2^. A substantial number of these compounds reveal cytotoxicity towards cancer cells, making them potential candidates for further drug development. By the beginning of 2024, 12 clinically-approved anticancer drugs derived from marine small molecules are available^3,4^.

**Figure 1:**
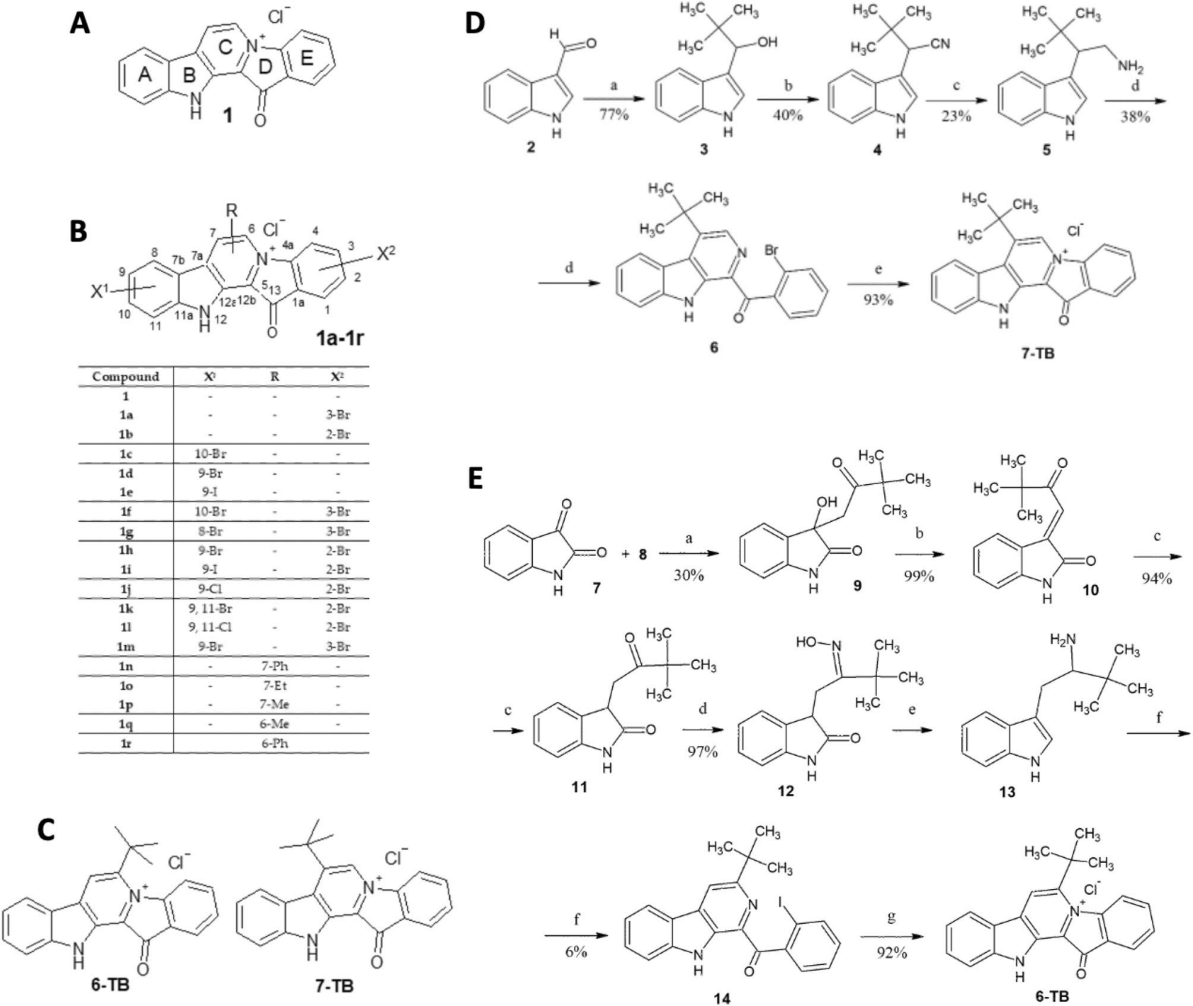
(**A**, **B**) Structures of fascaplysin (**1**, A) and some previously synthesized derivatives (**B**). (**C**) Structures of 6-TB and 7-TB. (**D**) Synthesis of 7-TB. Reagents and conditions: (**a**) Mg, *t*-BuCl, THF, r.t., then 3-formylindole, THF, reflux, 30 min; (**b**) NaCN, C_2_H_4_(OH)_2_, 120 °C, 50 min; (**c**) LiAlH_4_, Et_2_O, reflux, 1.5 h; (**d**) 2′-bromoacetophenone, I_2_, DMSO, 110 °C, 1 h, then incubation with corresponding tryptamine derivative, DMSO, 110 °C, 4 h; (**e**) 220 °C, 30 min, then HCl (aq). (E), Synthesis of 6-TB. Reagents and conditions: (**a**) KOH (0.6 equiv.), EtOH, 50 °C, 24 h; (**b**) HCl, CH_3_COOH, 75 °C, 2 h; (**c**) H_2_ (6 bar), 10% Pd/C, MeOH, r.t., 20 h; (**d**) NH_2_OH·HCl (4.2 equiv.), CH_3_COONa (4.1 equiv.), MeOH, 40 °C, 48 h; (**e**) 1) H_2_ (6 bar), PtO_2_, MeOH, r.t., 48 h, 2) NaBH_4_ (5.5 equiv.), BF_3_·OEt_2_ (6.0 equiv.), THF, 0 °C—r.t., 15 min, reflux, 2 h, then HCl (aq), reflux, 2 h; (**f**) 2′-iodoacetophenone, I_2_, DMSO, 110 °C, 1 h, then incubation with corresponding tryptamine derivative, DMSO, 110 °C, 4 h; (**g**) UV-irradiation, acetonitrile, − 5 °C, 3 × 0.5 h, then Na_2_CO_3_ (aq) and HCl (aq).

Fascaplysin (**1**, Fig. 1A) is a red cytotoxic pigment originally isolated from the marine sponge *Fascaplysinopsis* sp. in 1988^5^. This alkaloid possesses a 12*H*-pyrido[1,2-*a*:3,4-*b*′]diindole core^6^ and exhibits a broad spectrum of biological activities, including antiviral, antibacterial, antifungal, antimalarial, and antitumor activity^6–8^. In addition, fascaplysin has demonstrated a potent anticancer activity in in vitro and/or in vivo models of solid tumors (breast, colorectal, prostate, lung carcinoma, melanoma, and glioma) and hematological malignancies (acute myeloid leukemia) without significant side effects^8–15^. Even though antitumor activity of fascaplysin has been studied for the past 35 years, the precise mode of action remains unclear. To date, known mechanism of action of fascaplysin and its derivatives include inhibition of cyclin-dependent kinase 4 (CDK4)^6,7,16,17^, intercalation in double-stranded DNA, induction of TRAIL-signaling mediated apoptosis as well as antiangiogenic activity executed via downregulation of VEGF (reviewed in^18^). Notably, although CDK4/6 was described as a specific molecular target of fascaplysin (reviewed in^6^), recent studies suggested anticancer activity of this marine alkaloid to be executed in a CDK4-independent manner, thus indicating involvement of other pathways^12,19^. Indeed, fascaplysin has been reported to inhibit VEGFR2, TRKA, survivin, and HIF-1α^12^, therefore potentiating proapoptotic signaling. At the same time, fascaplysin-mediated activation of AKT and AMPK^20^ as well as activation of cytoprotective autophagy via inhibition of PI3K/AKT/mTOR signaling^13,21^ was shown to mitigate the cytotoxic effect of this drug^20,22,23^. In addition, the ability of fascaplysin to intercalate into DNA has been reported and confirmed by several groups^17,24^. This activity is conditioned by a five-ring coplanar backbone of the molecule and was suggested to be important for its cytotoxic activity^17,25^. Likewise, DNA-intercalating activity has been reported for several anticancer agents including anthracyclines, mitoxantrone, dactinomycin and was indeed shown to be one of the factors determining cytotoxic activity of these agents^26^.

Prostate cancer represents the most prevalent malignancy in men over the age of 50, with annual diagnoses exceeding 1.3 million cases, ranking it among the top five deadliest cancers globally^27^. In its initial stages, prostate cancer may not exhibit symptoms, and based on the tumor's biological characteristics and the stage of disease, patient may be managed through active surveillance, treatment with radical prostatectomy or radiation therapy with the intend to cure. In case of metastatic disease, treatment includes GnRH agonists in combination with new hormonal agents^28,29^. However, eventually all the patients develop resistance to hormonal treatment which results in castration-resistant prostate cancer (CRPC), which is considered practically incurable especially in its metastatic form (mCRPC)^30,31^. The therapeutic landscape for mCRPC is diverse and encompasses endocrine, chemotherapy, immunotherapy, and radioligand therapy^30^. Over the last years, advancements in mCRPC treatment have been marked by the approval of several strategies, including application of second generation of androgen receptor (AR)^32^ signaling inhibitors and PARP inhibitors (PARPi)^33^. Thus, docetaxel represents a primary chemotherapeutic option for CRPC, whereas abiraterone and enzalutamide, as second-generation anti-androgens also referred to as new hormonal agents (NHA) are approved for both mCRPC and hormone-sensitive prostate cancer^30^. A PARPi olaparib can be applied in mCRPC patients bearing homologous recombination-deficient tumors, e.g. with aberrant *BRCA1/2* gene^33^. Despite this progress, ultimate development of secondary drug resistance following long-term application of the therapeutics makes mCRPC eventually uncurable^34^. The mechanisms underlying this drug resistance are complex and not yet fully elucidated. Among others, overexpression of p-glycoprotein (p-gp) is one of the major mechanisms of anticancer therapy failure in prostate and other cancers, and is the main mechanism of resistance to docetaxel^35^. Additionally, development of tumors lacking androgen receptor (AR) or expressing permanently active AR variants (e.g. AR-V7) makes AR-targeting NHA therapy of prostate cancer inefficient^36^. Thus, despite considerable research efforts and ongoing studies of novel anticancer agents, the development of effective strategies to mitigate the progression of CRPC tumors and overcome their drug resistance remains an acute and still unmet medical need.

Recently, our group synthesized a library of 20 fascaplysin derivatives which comprised families substituted at cycles A, C and E^37^ (Fig. 1B). Further testing revealed their activity in prostate cancer including CRPC models. These previous results have suggested that the i) cycle C-derivatives are more cytotoxic compared to cycle A- and E-derivatives, and that ii) selectivity of fascaplysin derivatives towards tumor cells negatively correlates with their ability to intercalate into DNA^37^. Hence, to validate this hypothesis, in the current study we synthesized the cycle C-derivatives containing bulky substitutes, which are supposed to mitigate the DNA-intercalating activity of these molecules. Here, we report synthesis, biological evaluation and mechanism of action of these new fascaplysin derivatives 6-TB and 7-TB (Fig. 1C) in drug-resistant prostate cancer models.

## Results

### Synthesis of 6-*tert*-butylfascaplysin and 7-*tert*-butylfascaplysin

6-*tert*-butylfascaplysin (6-TB) and 7-*tert*-Butylfascaplysin (7-TB) were synthesized utilizing a modified two-step method reported by Zhu et al.^38^. To synthesize 7-TB, we performed Grignard reaction of 3-formylindole (**2**) with *tert*-butylmagnesium chloride (Fig. 1D, step **a**)^39^. Resulting product **3** was introduced to the substitution reaction with sodium cyanide to obtain nitrile **4** (Fig. 1D, step **b**)^40^. The reduction of compound **4** to tryptamine derivative **5** was carried out using LiAlH_4_ (Fig. 1D, step **c**). Then we applied a one-pot cascade coupling protocol that included the sequential iodination of corresponding acetophenone, the Kornblum oxidation of the intermediate in the presence of DMSO to phenylglyoxal, followed by its Pictet–Spengler condensation with tryptamine, and then by the oxidation of the intermediate product to generate 1-benzoyl-4-*tert*-butyl-β-carboline (**6**) (Fig. 1D, step **d**). Then product **6** was quaternizated at high temperature which resulted in 7-TB with 93% yield (Fig. 1D).

To synthesize 6-TB, we performed aldol condensation of isatin (**7**) with 3,3-dimethyl-2-butanone (**8**) (Fig. 1E, step **a**), followed by dehydration of the product **9** (Fig. 1E, step **b**) and the subsequent reduction of the double bond (Fig. 1E, step **c**) followed by oximation (Fig. 1E, step **d**). Tryptamine derivative **13** was synthesized through the catalytic hydrogenation over PtO_2_, followed by subsequent treatment with BH_3_·THF complex (Fig. 1E, step **e**)^24^. The tryptamine derivative **13** was converted to **14** using the same approach as was applied for **5** (see above). As the quaternization of 1-benzoyl-3-*tert*-butyl-β-carboline (**14**) could not be accomplished by exposure to high-temperature, we elaborated the modified UV irradiation-stimulated quaternization method to prepare of 6-TB^24^.

### Introduction of *tert*-butyl group reduces DNA-intercalating activity of fascaplysin

**Figure 2:**
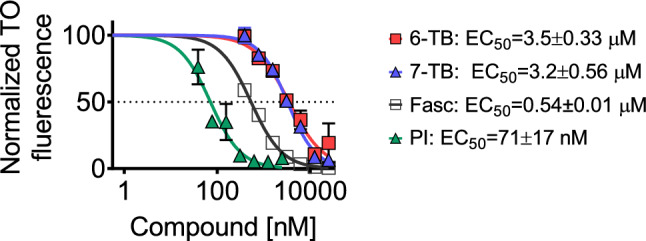
DNA intercalating activity of the synthesized derivatives. The activity was examined using a thiazole orange displacement assay. The incubation time was 7 min. Fascaplaysin (Fasc) was used as a reference substance and propidium iodide (PI) was used as a positive control. The experiment was performed in triplicates (number of biological replicates *n* = 3).

The introduction of a bulky *tert*-butyl group into the molecule was intended to decrease the DNA-intercalating activity of the fascaplysin derivatives. Therefore, we used a thiazole orange displacement assay to examine the ability of the synthesized compounds to intercalate into DNA. In line with our hypothesis, EC_50_s of both 6-TB and 7-TB were ~ sevenfold lower when compared to the mother fascaplysin molecule (Fig. 2).

### Activity and selectivity of 6-TB and 7-TB in human prostate cancer cells

The cytotoxic activity and selectivity of 6-TB and 7-TB were assessed using MTT assay and a panel of human prostate cancer and non-cancer cell lines. In these experiments we used malignant PC3, PC3-DR, 22Rv1, DU145, and LNCaP cells, as well as non-malignant PNT2, MRC-9, and HEK293 cell lines. LNCaP is a hormone-sensitive cell line, whereas other cancer cells exhibit resistance to androgen receptor (AR)-targeting therapies such as abiraterone and enzalutamide^41^. In 22Rv1 this resistance is attributed to the expression of AR splice variant 7 (AR-V7)^41^, which lacks an androgen binding site and therefore triggers permanent auto-activation of the AR pathway^42^. PC3, PC3-DR, and DU145 cells do not express AR and do not rely on androgens for their growth and proliferation^41^. The docetaxel-resistant PC3-DR cell line was generated by prolonged exposure of PC3 cells to increasing concentrations of docetaxel^43^.

Remarkably, 6-TB and 7-TB exhibited cytotoxic effects in all the tested cancer cell lines at low micro- and nanomolar concentrations, with the highest activity observed in 22Rv1 cells (Table 1). Notably, PC3-DR cells exhibit approximately a 50-fold decrease in sensitivity to docetaxel compared to their parental cell line (Table 1). Interestingly, the IC_50_s of both synthesized compounds in PC3-DR cells were only twofold higher compared to PC3 cells, indicating a minimal cross-resistance between docetaxel and synthesized fascaplysin derivatives (Table 1). The selectivity of the 6-TB and 7-TB towards cancer cells was comparable, but not higher than those of fascaplysin (Table 1). Finally, IC_50_ values generated using MTT and trypan blue exclusion methods were equal, indicating that suppression of metabolic activity (MTT test^44^) and disruption of cellular membrane integrity (trypan blue exclusion methods^45^) happen rather simultaneously (Table 1). Thus, 6-TB and 7-TB did not possess any specific antimetabolic effect as it has been demonstrated for some other previously reported derivatives^46^.

**Table 1:** Cytotoxicity of 6-TB and 7-TB in human prostate cancer cells.

IC_50_, MTT assay									IC_50_, Trypan blue staining assay	Selectivity index, Cancer vs non-cancer cells
Drug	Cancer cells					Non-cancer cells			22Rv1 cells	
	PC3	PC3-DR	22Rv1	DU145	LNCaP	PNT2	MRC-9	HEK293		
6-TB (µM)	1.1 ± 0.076	2.26 ± 0.16	0.58 ± 0.073	1.73 ± 0.172	1.33 ± 0.24	1.82 ± 0.488	1.37 ± 0.424	0.348 ± 0.09	0.474 ± 0.133	0.99
7-TB (µM)	0.223 ± 0.12	0.41 ± 0.023	0.105 ± 0.098	0.336 ± 0.102	0.297 ± 0.039	0.276 ± 0.091	0.31 ± 0.007	0.16 ± 0.025	0.109 ± 0.059	1.02
Fascaplysin (µM)	0.766 ± 0.126	1.61 ± 0.92	0.242 ± 0.081	0.798 ± 0.054	0.409 ± 0.02	0.457 ± 0.075	0.89 ± 0.046	0.458 ± 0.19	0.335 ± 0.093	1.09
Docetaxel (nM)	7.49 ± 7.09	324.9 ± 21.7	0.6 ± 0.12	2.2 ± 0.6	5.06 ± 0.58	> 500	> 500	11.49 ± 3.22	0.41 ± 0.22	4.95

Cells were treated with the investigated compounds for 48 h. Values are represented as mean ± standard deviation. The experiment was performed in triplicates (number of biological replicates *n* = 3).

We further investigated the mode of action of 6-/7-TB in prostate cancer cells. 22Rv1 cells, which represent a drug-resistant prostate cancer and at the same time are highly sensitive to the synthesized alkaloids, were selected as the main model for further experiments. In addition, we mainly focused on 7-TB derivative as this compound was ~ fivefold more potent than 6-TB and therefore was considered more promising.

**Figure 3:**
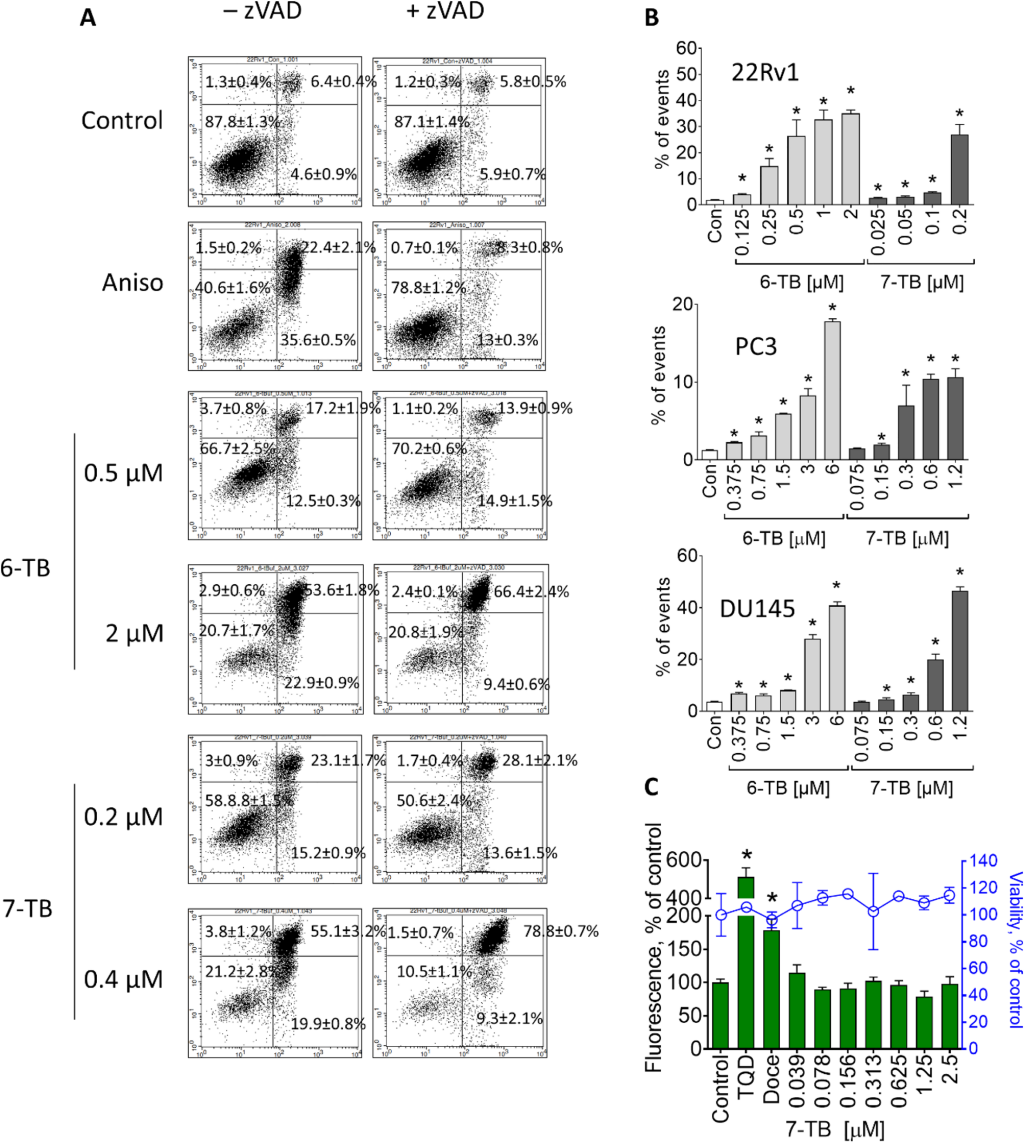
Effect of synthesized compounds on apoptotic markers and p-gp activity. (**A**) Annexin-V-FITC/PI double staining of apoptotic cells. Cells were treated with indicated concentrations of the drugs for 48 h with or without pre-treatment with 100 µM of pan-caspase inhibitor zVAD. Then the cells were harvested and analyzed using flow cytometry an annexin-V-FITC/PI double staining. Cells positive with annexin-V, PI, or both were considered as apoptotic. Anisomycin (Aniso, 10 µM) was used as a positive control. (**B**) DNA fragmentation analysis. 22Rv1, PC3 or DU145 cells were treated with tested compounds for 48 h. Then the cells were harvested, stained with PI and analyzed using flow cytometry. Cells appeared as sub-G1 population were assumed to contain fragmentated DNA. (**C**) Effect on the activity of p-glycoprotein (p-gp). PC3-DR cells were treated with 7-TB for 30 min, and subsequently incubated with calcein-AM solution for 15 min and the green fluorescence intensity was measured. P-gp inhibitor tariquidar (TQD, 50 nM) and p-gp substrate docetaxel (Doce, 100 nM) were used as positive controls. Additionally, the cytotoxic effect of the drugs was quantified using MTT assay under the same experimental conditions (denoted as a blue graph). Significant deviation from the control is marked with an asterisk (*), indicating *p*-value < 0.05, one-way ANOVA. All the experiments were performed in triplicates (number of biological replicates *n* = 3).

We further assessed induction of such apoptotic hallmarks as externalization of phosphatidylserine to the outer membrane as well as fragmentation of DNA. Notably, a dose-dependent upregulation of both above-mentioned markers was detected in 22Rv1 cells following 48 h of drug exposure (Fig. 3A, B). However, co-treatment with pan-caspase inhibitor zVAD could not mitigate the cytotoxic effect of 7-TB, whereas pro-apoptotic effect of anisomycin (positive control, an established inducer of the classical caspase-dependent apoptosis) was effectively inhibited (Fig. 3A). These results indicate an apoptosis-like but caspase-independent character of the cell death induced by the synthesized alkaloids.

### 7-TB is not a substrate or inhibitor of p-glycoprotein

Another important observation was comparable cytotoxicity of the drugs in both docetaxel-sensitive PC3 and docetaxel-resistant PC3-DR cells (Table 1). A key factor of resistance of PC3-DR cells to docetaxel is overexpression of p-glycoprotein (p-gp, MDR1). P-gp acts as a molecular pump expelling certain cytotoxic molecules (including docetaxel) out of the cancer cell. Elevated p-gp expression results in increased IC_50_ for docetaxel. i.e. drug-resistance of cells^35^. Comparable cytotoxicity in PC3 and PC3-DR cells suggests that the investigated compounds are not p-gp substrates (Table 1). Hence, we then assessed the impact of 7-TB on p-gp activity using a calcein-AM exclusion assay. In this assay, calcein-AM, a non-fluorescent dye, passively enters cells and is converted into fluorescent calcein by cellular esterases. Calcein and calcein-AM, both substrates of p-gp, are quickly expelled from cells with high p-gp expression, leading to reduced green fluorescence. The addition of p-gp inhibitors like tariquidar hampers p-gp function, resulting in an accumulation of calcein and thus increased fluorescence, making it a useful indicator of p-gp activity (Fig. 3C). Similar effects have been observed when p-gp substrate docetaxel is introduced. The reason for this is the concurrent binding of the p-gp substrates docetaxel and calcein to the transporter (Fig. 3C). At the same time, incubation of PC3-DR cells with 7-TB did not result in any significant alteration of calcein-AM excretion (Fig. 3C), indicating 7-TB is neither p-gp substrate nor p-gp inhibitor.

### Effect of 7-TB on the kinome of prostate cancer cells

Protein kinases regulate function of various protein through phosphorylation^47^. Among others, serine/threonine kinases (STK) play an important role in a number of critical cellular processes including survival and apoptosis^47^. STK are targets for several FDA-approved anticancer drugs, such as trametinib, selumetinib, palbociclib, and others^47^. Therefore, we evaluated the effect of 7-TB on kinome of prostate cancer cells using a functional kinome profiling. Functional kinome profiling was performed using the PamTechnology^48^ (http://www.pamgene.de, Fig. 3) which is a peptide based array system allowing to predict STKs whose activity is affected by the drug. In this assay a set of on-chip immobilized peptides known to be STK substrates is exposed to the cell lysate. Alteration of the peptide phosphorylation profile suggests STKs which activity was altered upon the treatment^48^.

**Figure 4:**
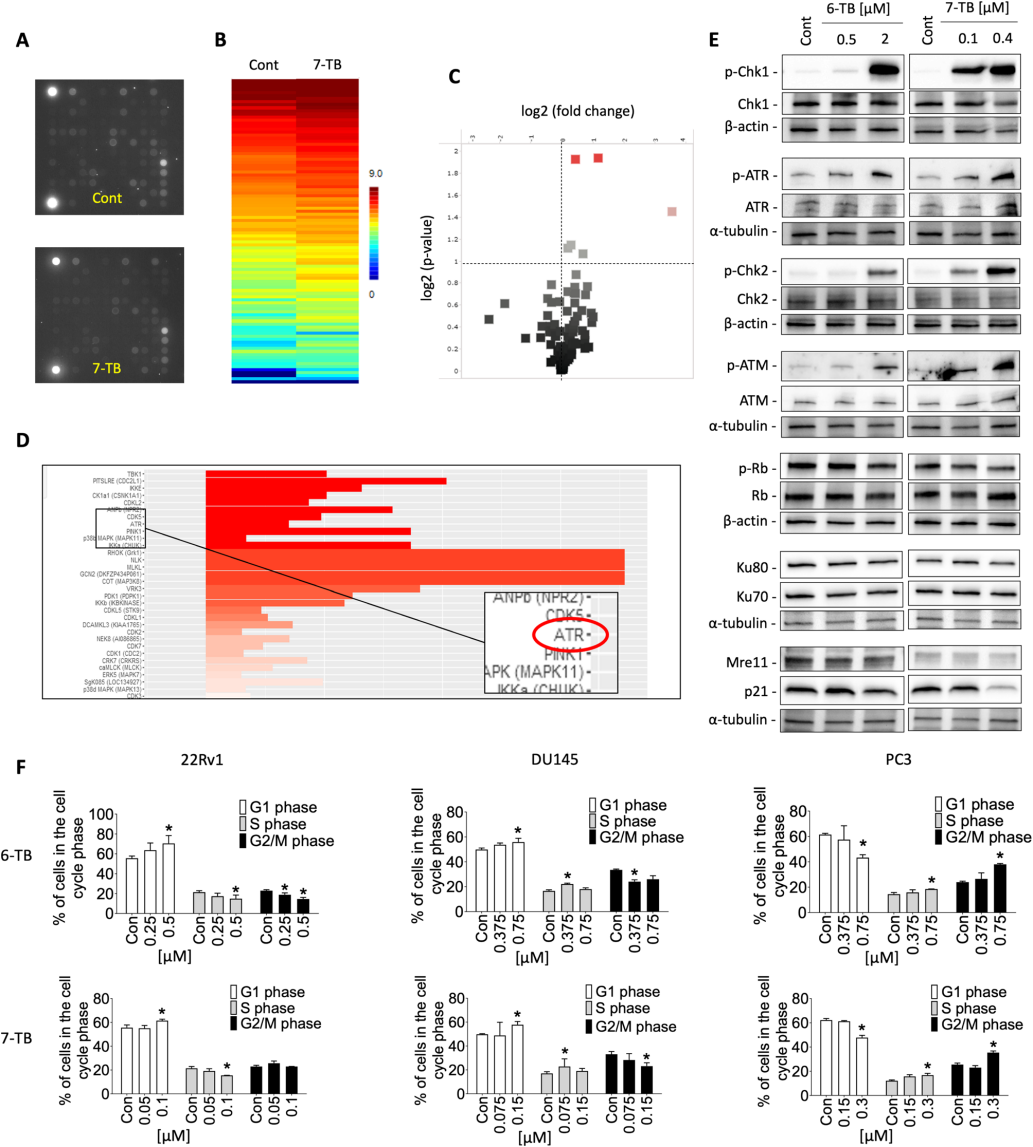
Functional kinome profiling of serine/threonine kinases and results validation. 22Rv1 cells were incubated with 7-TB for 2 h followed by lysis, protein extraction and analysis using specific STK-PamChips^®^. (**A**, **B**, **C**) Representative photos of the STK-PamChips^®^, the heatmap of the 99 analyzed peptides (S100_log transformed values) (**B**) volcano-plot (**C**) of affected peptides. Red dots represent peptide substrates which phosphorylation significantly increased in 7-TB treated samples versus control. Dotted line indicate log2(p) > 1.3. (**D**) Upstream kinase analysis, predicting kinases to be affected by 7-TB in 22Rv1 cells. Bars represent normalized kinase statistic, (log2) > 0 indicate increase of kinase activity in cells treated with 7-TB. Statistically significant changes (specificity score, log2 > 1.3) are indicated by white to red bars. (**E**) Western blotting analysis of protein expression. The original full-size blots are represented in Supplementary Figure S2. 22Rv1 cells were treated with investigated drugs at the indicated concentrations. β-actin or α-tubulin were used as loading controls. (**F**) Cell cycle analysis. 22Rv1, PC3, or DU145 cells were treated with tested compounds for 48 h. Cells were harvested, stained with PI and analyzed using a flow cytometry. Statistically significant difference from control is indicated as: *p < 0.05 (ANOVA). Kinome profiling experiments were performed in triplicates (number of technical replicates *n* = 3); other experiments were performed in triplicates (number of biological replicates *n* = 3).

In order to minimize interference from the secondary cell death-related events, we applied a short-term 2 h drug exposure. The findings, represented as log2 of signal intensity per peptide, compared control and treated groups (Fig. 4A, B, C). Further upstream kinase analysis predicting STKs potentially affected by 7-TB treatment suggested an increase in the activity ATR kinase (Fig. 4D).

### Validation of kinome profiling results

To further validate the predictions of the functional kinome profiling we performed Western blotting analysis of the kinases predicted to be induced under the treatment. CHK1 kinase is a well-known direct and specific downstream target of ATR^49^. Thus, we examined the expression of proteins involved in ATR/CHK1 pathway, as well as of the proteins belonging to another closely interconnected ATM/CHK2 pathway^49^. Remarkably, we detected an increased phosphorylation of ATR and CHK1, as well as ATM and CHK2 kinases, indicating activation of both pathways (Fig. 4E). ATR/CHK1 pathway is usually activated in response to single-stranded DNA breaks (SSB) and/or replication stress, leading to S- and G2/M-cell cycle arrest^50^, further DNA repair, and ultimately cell survival^49^. In contrast, CHK2 is commonly triggered by double-strand breaks (DSBs) in DNA, initiating G1 arrest so that the cells bearing damaged DNA are prevented from entering S-phase^50^. Hence, we further examined the effect of 6- and 7-TB on cell cycle progression. Interestingly, both compounds induced G1 cell cycle arrest in *BRCA1/2*-deficient 22Rv1 and DU145 cells, whereas *BRCA1/2*-wild type PC3 cells revealed G2/M arrest (Fig. 4F)^51–54^.

### Activation of ATR/CHK1 axis is a prosurvival factor in cellular response to the drug treatment

**Figure 5:**
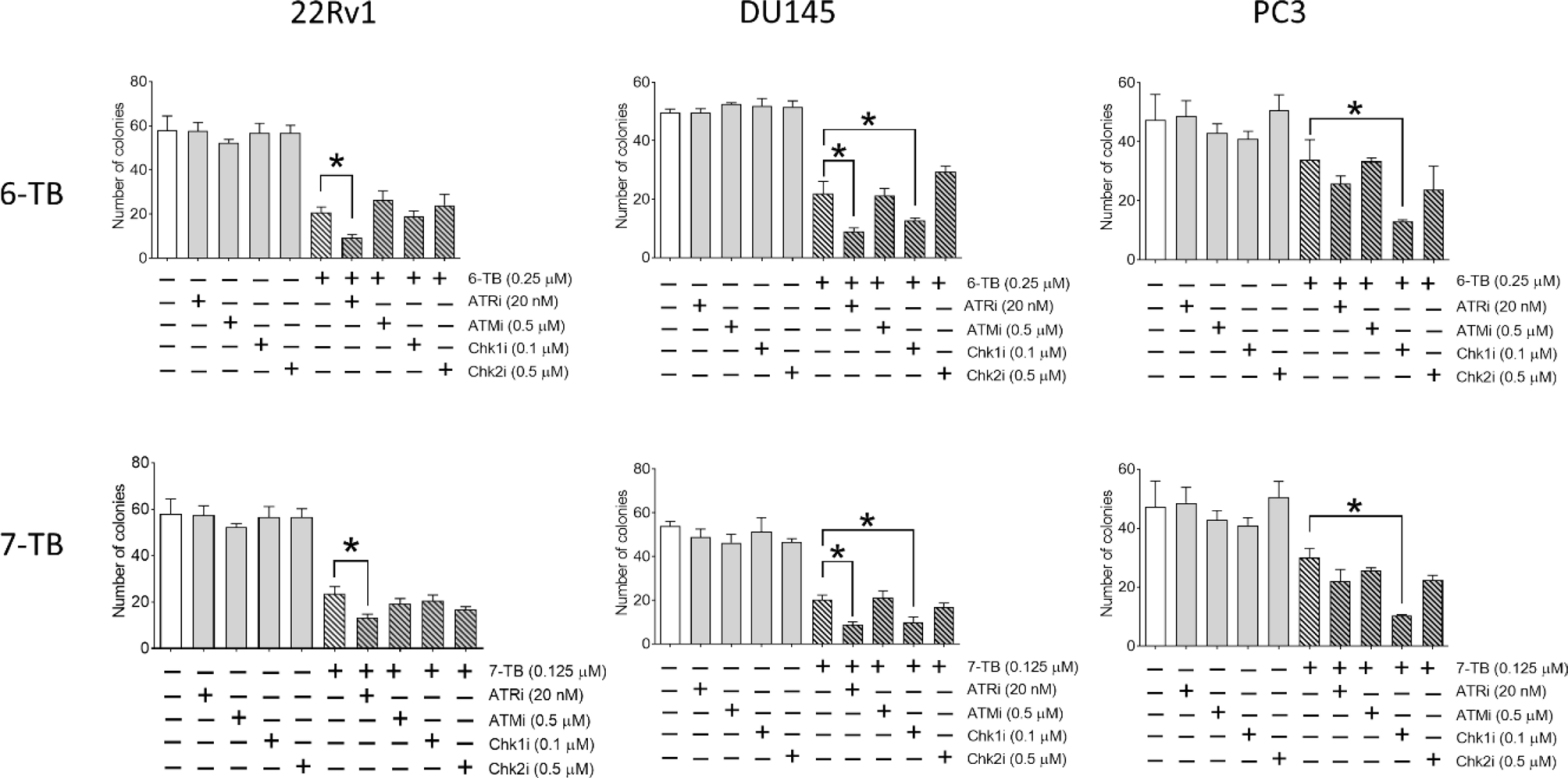
Effect of DNA damage response inhibitors on the anticancer activity of 6-TB and 7-TB. Effect was examined using a colony formation assay. Cells were seeded into 6-well plates and treated with tested compounds with or without specific inhibitor at the indicated concentrations for 10–14 days. The colonies were fixed, stained and counted. The inhibitors used are indicated as ATRi (VE-822), Chk1i (LY2603618), ATMi (KU-60019), Chk2i (PV1019). Statistically significant difference between group treated with 6-/7-TB alone and group co-treated with either inhibitor is indicated as: *p < 0.05 (ANOVA); otherwise, the difference is not statistically significant. Experiments were performed in triplicates (number of biological replicates *n* = 3).

As a next step, we performed a functional assay to establish the role of ATR/CHK1 and ATM/CHK2 pathways in the cell death induced by 6-TB and 7-TB in three human prostate cancer cell lines. Thus, we evaluated cytotoxic effects of the investigated compounds in the presence of specific inhibitors of either kinase at their non-cytotoxic concentrations. The viability was evaluated using colony formation assay. In line with kinomics analysis results, we observed that inhibition of ATR/CHK1 pathway increases activity of the synthesized drugs, whereas inhibition of ATM/CHK2 did not alter cytotoxicity significantly (Fig. 5). This effect has been observed for both compounds in both *BRCA1/2*-mutant and -wild type cells. At the same time, effect of ATR inhibition hampered 6-/7-TB activity in *BRCA1/2*-mutant 22Rv1 and DU145 cells, but not in *BRCA1/2*-wild type PC3 cells^51–54^. On the other hand, inhibition of CHK1 kinase, a downstream target of ATR, but not inhibition of ATR kinase itself, resulted in significantly increased cytotoxic activity of 6-/7-TB in PC3 cells (Fig. 5).

### Induction of DNA double-strand breaks by 6- and 7-TB

**Figure 6:**
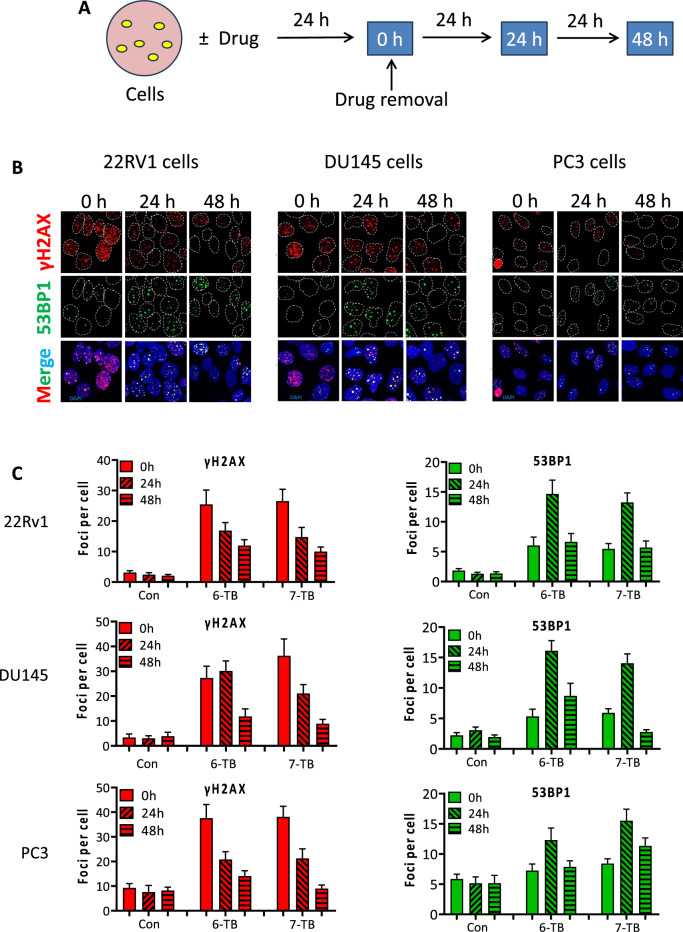
Examination of DNA double-strand breaks formation and repair following the treatment. (**A**) The treatment schema. 22Rv1, DU145 and PC3 cells were treated with the investigated drugs for 24 h followed by drug-free growth and staining 0–48 h. (**B**, **C**) Representative pictures (**B**) and quantification (**C**) of γH2AX (red) and 53BP1 (green) foci staining. 22Rv1, DU145 and PC3 cells were analyzed 0 h, 24 h, and 48 h following the treatment. For each treatment group a minimum of 100 cells were analyzed. Experiments were performed in duplicates (number of biological replicates *n* = 2) and at least 20 microscopic fields per replicate were analyzed.

Both ATR/CHK1 and ATM/CHK2 pathways can be activated in response to DNA DSBs and/or replication stress^50^. Therefore, we examined formation of both DSB and replication fork stalling as well as dynamics of these processes. Therefore, we monitored the number of γH2AX and 53BP1 foci as markers of DSB in 22Rv1, DU145 and PC3 cells after different time points following 24 h incubation with 6-TB or 7-TB (Fig. 6A). Intriguingly, time dependent analysis revealed distinct kinetics for both DSB markers. γH2AX exhibited the highest number immediately after the 24 h incubation period (0 h time point, Fig. 6B, C). Subsequently, this number began to decline in a time-dependent manner after the removal of the drug (Fig. 6C). On the other hand, 53BP1 foci demonstrated a modest increase after the 24 h incubation period (0 h time point), followed by a profound increase 24 h post-removal of the drug (Fig. 6C). Upon induction of DSBs, H2AX is phosphorylated at S-139, generating the γH2AX, rapidly by ATM/ATR, depending on the cell cycle phase^50^. γH2AX then serves as a docking platform for the recruitment of other downstream DDR factors, such as 53BP1. Therefore, the kinetics of both markers (i.e. γH2AX and 53BP1) should basically coincide with each other. Since we observed a much higher γH2AX foci number than the 53BP1 recruited to the damage sites immediately after drug incubation, and considering that γH2AX also marks non-DSB damages, such as replication stress^55^, we thus speculate that the main types of DNA damage induced by the drugs are replication stress.

### Induction of replication stress by 6-and 7-TB

**Figure 7:**
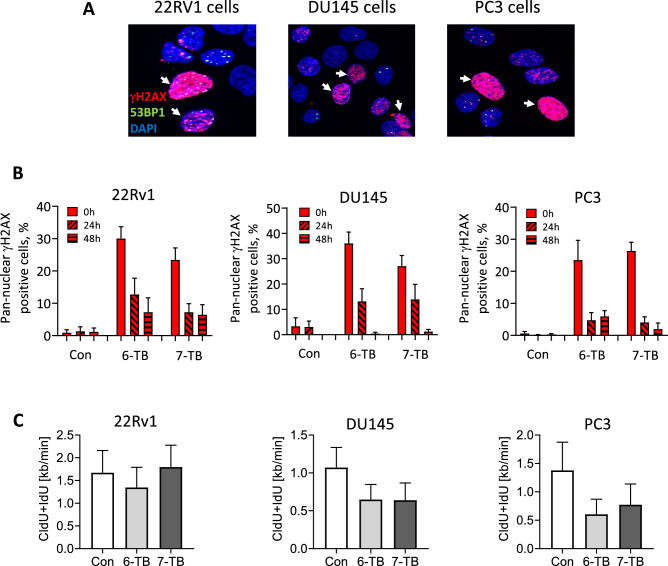
Evaluation of treatment-induced replication stress. 22Rv1, DU145 and PC3 cells were treated with the individual drugs at given concentrations for 24 h. (**A**, **B**) Representative pictures (**A**) and quantification (**B**) of the immunofluorescence staining for γH2AX (red) and nuclei (DAPI, blue). The staining was performed 0 h, 24 h, and 48 h after the treatment. Cells with a pan-nuclear γH2AX staining were assumed to undergo a replication stress. (**C**) Frequency distribution of DNA fiber lengths. The cells were treated with the drugs for 24 h followed by a consecutive labeling with CldU and IdU. Spread DNA was labeled with appropriate antibodies and incorporated CldU and IdU were detected. Data are represented as means ± SD of DNA fiber length measured as a sum of CldU and IdU fibers. Experiments were performed in duplicates (number of biological replicates *n* = 2) and at least 20 microscopic fields per replicate were analyzed.

Previously, it has been demonstrated that excessive replication stress induces pan-nuclear γH2AX^56^. Therefore, we next sought to monitor nuclei with pan-nuclei γH2AX staining as a marker of replication fork stress upon treatment with the drugs. We could demonstrate a significant increase in the number of pan-nuclear γH2AX positive cells following a 24 h incubation with 6-/7-TB (Fig. 7A, B). This number declined in a time-dependent manner, reaching 1–20% after 48 h post drug removal compared to 0 h time point. This data implies replication stress accumulation upon treatment by 6-TB or 7-TB. To address this issue in more details, we employed DNA fiber assay to study DNA replication and measure replication fork speed upon drug treatment. This technique assesses the dynamics of DNA replication at the single-molecule level. In this assay the DNA fibers are consequently stained with fluorescent markers (e.g. CldU and IdU) that label newly synthesized DNA, allowing visualization of DNA replication and measurement of replication fork speed. Our analysis revealed a significant slowdown in replication fork speed in all cell lines investigated upon treatment with either 6-TB or 7-TB (Fig. 7C). Overall, these data suggest that both 6-TB and 7-TB exhibit their antitumor activity by impeding replication dynamics, causing replication stress. This observation explains the higher numbers of γH2AX observed 24 h after drug treatment. If collapsed, these stresses would lead to the accumulation of DSBs, explaining the increased number of the DDR factor 53BP1 observed 48 h after drug removal, most of which coincide with γH2AX.

## Discussion

Fascaplysin alkaloids are a family of bioactive marine-derived compounds which have previously shown a potent anticancer activity both in vitro and in vivo. Our group has recently reported synthesis of a library of fascaplysin derivatives with substitutes introduced to cycles A, C and E of the 12*H*-pyrido[1–2-*a*:3,4-*b*′]diindole core^37^. Interestingly, in that study we observed that the selectivity towards cancer cells anticorrelated with DNA intercalating activity of the fascaplysin derivatives^37^. Hence, we speculated that introduction of a bulky group further decreases the DNA intercalating activity and therefore may increase selective cytotoxicity in cancer cells. Hence, we synthesized two new *tert*-butyl fascaplysin derivatives, namely, 6- and 7-*tert*-butylfascaplysins (6-TB and 7-TB, respectively). These compounds had significantly lower ability to intercalate into double-strand DNA compared to the mother unsubstituted alkaloid. Both, 6-TB and 7-TB showed a selectivity index comparable to fascaplysin. Notably, 7-TB was the most active derivative and was twice more cytotoxic than the natural alkaloid fascaplysin. These observations indicate a complex nature of structure–activity relationships within the fascaplysin family, suggesting that cytotoxicity and selectivity of these alkaloids are influenced by multiple factors. In future, selectivity of fascaplysin may be improved using other strategies such as covalent conjugation with monoclonal antibodies (i.e. antibody–drug conjugates, ADC) at the C-7 position of fascaplysin core resembling 7-TB. Here, conjugation with either glucose or folic acid residues for the Warburg effect targeting or folate targeting, respectively, may be a promising strategy. Importantly, the synthetic methods developed by us allow an introduction of another bulky and targeted moiety in the 6 and 7 position of facsaplysin scaffold.

Despite various treatment options such as androgen receptor-directed therapies, taxane-based chemotherapy, PSMA radioligand therapy, radium-223 and PARP inhibitors, prostate cancer in the castration-resistant situation takes a fatal course. P-gp overexpression as well as loss, mutation or variant expression of AR are among the main mechanisms of prostate cancer therapy resistance. P-gp acts as a molecular pump excreting taxanes and other therapeutics out of the cell thereby mitigating their cytotoxic effect and limiting the efficacy of alternative therapeutical options^35^. Therefore p-gp inhibitors as well as cytotoxic agents lacking affinity to p-gp are of particular interest. It is important to note 6- and 7-TB were not identified as substrates of p-gp and in line with this demonstrated a potent cytotoxicity in both taxane-sensitive p-gp^low^ PC3 and taxane-resistant p-gp^high^ PC3-DR cells with no cross-resistance observed. Moreover, the synthesized compounds exhibited comparable activity in NHA-sensitive LNCaP cells (AR^+^), partially sensitive 22Rv1 cells (AR^+^ and AR-V7^+^), and insensitive PC3, PC3-DR, and DU145 cells (AR^−^). These results indicate 6-/7-TB to be promising candidates for therapy of the prostate cancer refractory to taxanes and/or antihormonal agents.

Based on our results, we propose that the mechanism of action identified for synthesized derivatives is the induction of replication stresses which eventually result in and the accumulation of DNA DSBs. This effect was observed as early as 2 h after exposure and prior to any other alteration of cellular viability. It is therefore suggested to be one of the primary effects of the drugs. In turn, DSBs being highly toxic DNA lesions further lead to caspase-independent apoptosis-like cell death. Supportive to our model, we reported a much slower replication rate in different prostate cancer cells upon treatment by either 6-TB or 7-TB, leading to accumulation of pan-nuclear γH2AX staining and later to accumulation of DSBs as evidenced by increased number of 53BP1 foci. It is important to note that the induced replication stress was not related to DNA intercalating activity of the drugs, as the latter was abolished by the introduction of a bulky *tert*-butyl group into the molecule. This mode of action differs from fascaplysin as well as some other established anticancer drugs, which highlights the potential of 6-/7-TB and their derivatives as novel promising therapeutic agents.

In addition, functional kinome profiling screening approach and further validation by immunoblotting revealed the existence of kinase-mediated cellular responses to the treatment with investigated alkaloids. Specifically, we observed activation of ATR/CHK1 and ATM/CHK2 pathways, confirming the induction of replication associated stresses and DSBs. Interestingly, inhibition of the ATR/CHK1 pathway (e.g. using ATRi) further potentiated the cytotoxic effects of 6-/7-TB. This finding further consolidates our hypothesis that the cytotoxic effects are exerted by these drugs mainly during S/G2 cell cycle phase.

The effectiveness of the synthesized alkaloids also appears to be influenced by the *BRCA1/2* gene status. Mutations of *BRCA1/2* impair homologous recombination, which is a critical pathway for repairing of DSB. Therefore, *BRCA1/2*-mutant cells are known to be more dependent on the ATR/CHK1 pathway for maintaining their genome stability^57^. Hence, *BRCA1/2* impairment leads to increased replication stress and reliance on alternative repair pathways, thereby elevating the importance of the ATR/CHK1 axis^57^. This may also explain higher sensitivity of BRCA2-mutant 22Rv1 and DU145 cells to combination of 6-/7-TB and ATR inhibitors in comparison to *BRCA1/2*-wild type PC3 cells, pointing to a potential avenue for personalized medicine. Our results indicate possible beneficial effect of the combinational application with ATR/CHK1 inhibitors like berzosertib, ceralasertib and prexasertib. Additionally, taking into account the above-described DNA-targeting activity of 6-/7-TB, combination with PARP inhibitors (PARPi) may be another possible strategy for further development of these drugs. Moreover, it has been previously shown that combination with castration-inducing agents makes prostate cancer cells more sensitive to DNA-damaging agents^58^. Therefore, combinations with either GnRH agonists or with AR-targeting NHA is of specific interest for prostate cancer therapy and may also be considered in the future drug testing.

## Conclusion

In summary, we synthesized and evaluated two new members of the fascaplysin alkaloid family, 6- and 7-*tert*-butylfascaplysins. Both compounds were highly cytotoxic in both drug-sensitive and -resistant prostate cancer cells, and 7-*tert*-butylfascaplysins had superior activity compared to fascaplysin. The drugs had a decreased DNA intercalating activity compared to fascaplysin, and comparable selectivity towards cancer cells. The mechanism of action was identified as induction of replication stress that eventually converted to the toxic DNA DSBs, resulting in caspase-independent apoptosis-like cell death. Our results shed a light on new aspects of mechanism of action of fascaplysin and its derivatives, and contribute to further understanding of the complex structure–activity relationships within this molecular family.

## Materials and methods

### Chemistry

All of the starting materials are commercially available. Commercial reagents were used without any purification. For UV irradiation, the high-pressure mercury UV lamp DRT-1000 was used. The products were isolated by MPLC: Buchi B-688 pump; glass column C-690 (15 × 460 mm) with silica gel (particle size 0.015–0.040 mm); and UV detector Knauer K-2001. The analytical examples were purified by the Shimadzu HPLC system (model: LC-20AP) equipped with a UV detector (model: SPD 20A), using a Supelco C18 (5 µm, 20 × 250 mm) column using the MeOH:H_2_O (20:80, 50:50, 70:30) mobile phase by isocratic elution at a flow rate of 15 mL/min. The NMR spectra were recorded with an NMR instrument operating at 400 MHz (^1^H) and 100 MHz (^13^C). Proton spectra were referenced to TMS as an internal standard and, in some cases, to the residual signal of used solvents. Carbon chemical shifts were determined relative to the ^13^C signal of TMS or the used solvents. Chemical shifts are given on the *δ* scale (ppm). Coupling constants (J) are given in Hz. Multiplicities are indicated as follows: s (singlet), d (doublet), t (triplet), q (quartet), m (multiplet), or br (broadened). The original NMR spectra of the relative compounds can be found in Supplementary Figure S1. High-resolution mass spectra (HRMS) were obtained with a time-of-flight (TOF) mass spectrometer (model Agilent TOF 6210) equipped with an electrospray source at atmospheric pressure ionization (ESI). The purity of the synthesized compounds was established based on the NMR and HRMS spectral information.

*1-(1H-Indol-3-yl)-2,2-dimethylpropan-1-ol (3)*

Compound **2** was obtained by a known method^39^. For the synthesis, 3.0 mmol of 3-formylindole was taken with a product yield of 77%. ^1^H NMR (400 MHz, CDCl_3_): *δ* 8.15 (br. s, 1H), 7.72 (d, J = 8.0 Hz, 1H), 7.37 (d, J = 8.1 Hz, 1H), 7.20 (td, J = 7.5, 1.0 Hz, 1H), 7.16 (d, J = 2.4, 1H ), 7.12 (m, 1H), 4.83 (d, J = 2.0 Hz, 1H), 1.83 (d, J = 2.2 Hz, 1H ), 1,02 (s, 9H). ^13^C NMR (100 MHz, CDCl_3_): *δ* 135.8, 127.0, 122.3, 121.9, 120.2, 119.6, 118.3, 111.0, 76.6, 36.4, 26.1.

*2-(1H-Indol-3-yl)-3,3-dimethylbutanenitrile (4)*

Compound **3** was obtained by a previously published method^40^. Sodium cyanide was used to carry out the substitution reaction. For the synthesis, 3.4 mmol of 1-(3-indolyl)-2,2-dimethylpropanol was taken with a product yield of 40%. ^1^H NMR (400 MHz, CDCl_3_): *δ* 8.26 (br. s, 1H), 7.63 (d, J = 8.1 Hz, 1H), 7.40 (d, J = 8.1 Hz, 1H), 7.23 (m, 2H), 7.17 (m, 1H), 3.9 (s, 1H), 1.13 (s, 9H). ^13^C NMR (100 MHz, CDCl_3_): 135.8, 126.7, 124.1, 122.4, 120.9, 120.1, 119.1, 111.3, 108.9, 40.8, 35.7, 27.6.

*2-(1H-Indol-3-yl)-3,3-dimethylbutylamine (5)*

Compound **3** was obtained by a previously published method^40^. For the synthesis, 1.3 mmol of 2-(3-indolyl)-3,3-dimethylbutyronitrile was taken with a product yield of 23%. Due to instability, the product was introduced to the subsequent reaction without further purification. MS–ESI, m/z: [M + H]^+^ 217.11.

*3-(3,3-Dimethyl-2-oxo-butyl)-3-hydroxy-indolin-2-one (9)*

Pinacoline (**6**) (6.00 g, 59.9 mmol) and NaOH (1.15 g, 28.8 mmol) in 5 mL of H_2_O were successively added to a suspension of isatin (**5**) (5.00 g, 34.0 mmol) in EtOH (100 mL). Next, the reaction mixture was stirred on a magnetic stirrer heated to 50 °C. The reaction mixture was kept at a constant weak alkaline pH for 24 h. The progress of the reaction was monitored by TLC. After the appearance of traces of the by-product, the reaction was complete. Then, the mixture was evaporated under reduced pressure, and the residue was recrystallized from H_2_O. The crystals were filtered off, washed with cold H_2_O and dried. The obtained product was light beige crystals (2.30 g, 30%).

^1^H NMR (400 MHz, CDCl_3_): *δ* 8.46 (s, 1H), 7.30 (d, *J* = 7.4 Hz, 1H), 7.24 (td, *J* = 7.8, 1.0 Hz, 1H), 7.02 (td, *J* = 7.5, 0.6 Hz, 1H), 6.88 (d, *J* = 7.7 Hz, 1H), 4.82 (s, 1H), 3.33 (d, *J* = 17.6 Hz, 1H), 3.05 (d, *J* = 17.6 Hz, 1 H), 1.08 (s, 9H). ^13^C NMR (100 MHz, CDCl_3_): δ 215.3, 178.6, 140.7, 130.4, 129.9, 124.0, 123.0, 110.5, 74.8, 44.6, 42.6, 25.9. HRMS-ESI, *m/z*: [M + H]^+^ calculated for C_14_H_18_NO_3_^+^ 248.1281, obtained 248.1291.

*3-(3,3-Dimethyl-2-oxo-butylidene)indolin-2-one (10)*

Compound **7** (2.00 g, 8.1 mmol) was dissolved in 5 mL of CH_3_COOH, and 4 drops of HCl (aq) were added to the solution. The reaction mixture was stirred on a magnetic stirrer heated to 75 °C. The progress of the reaction was monitored by TLC. Then, the mixture was poured into H_2_O and neutralized with NaHCO_3_; the solution was extracted with EtOAc (3 × 20 mL); and the extract was dried and evaporated under reduced pressure. The obtained product was orange crystals (1.84 g, 99%).

^1^H NMR (400 MHz, CDCl_3_): *δ* 8.36 (d, 7.8 Hz, 1H), 8.21 (br. s, 1H), 7.46 (s, 1H), 7.32 (td, *J* = 7.7, 0.9 Hz, 1H), 7.02 (td, *J* = 7.7, 0.8 Hz, 1H), 6.86 (d,* J* = 7.7 Hz, 1H), 1.29 (s, 9H). ^13^C NMR (100 MHz, CDCl_3_): *δ* 206.5, 169.5, 143.1, 136.1, 132.6, 128.1, 125.7, 122.9, 120.7, 110.0, 44.8, 26.2. HRMS-ESI, *m/z*: [M + H]^+^ calculated for C_14_H_16_NO_2_^+^ 230.1176, obtained 230.1193.

*3-(3,3-Dimethyl-2-oxo-butyl)indolin-2-one (11)*

Compound **8** (0.98 g, 4.3 mmol) was dissolved in 30 mL of MeOH. A catalytic amount of 10% Pd/C was added to the solution. The reaction was carried out in a hydrogen atmosphere (6 bar). The mixture was stirred at room temperature for 18 h. The bright orange color of the solution disappeared. The mixture was then poured into H_2_O and extracted with EtOAc (3 × 20 mL). The extract was dried and evaporated under reduced pressure. The obtained product was light yellow crystals (0.93 g, 94%).

^1^H NMR (400 MHz, CDCl_3_): *δ* 8.30 (br. s, 1H), 7.19 (t, *J* = 7.7 Hz, 1H), 7.09 (d, *J* = 7.4 Hz, 1H), 6.97 (td,* J* = 7.3, 0.6 Hz, 1H), 6.88 (d, *J* = 7.8 Hz, 1H), 3.91 (dd, *J* = 8.5, 3.2 Hz, 1H), 3.31 (dd, *J* = 18.4, 3.3 Hz, 1H), 3.00 (dd, *J* = 18.3, 8.6 Hz 1H), 1.17 (s, 9H). ^13^C NMR (100 MHz, CDCl_3_): *δ* 212.8, 180.0, 141.4, 129.7, 128.0, 124.2, 122.4, 109.6, 43.9, 41.4, 38.1, 26.4. HRMS-ESI, *m/z*: [M + H]^+^ calculated for C_14_H_18_NO_2_^+^ 232.1332, obtained 232.1332.

*3-[2-Hydroxyimino-3,3-dimethyl-butyl]indolin-2-one (12)*

Finely ground NH_2_OH·HCl (1.50 g, 21.5 mmol) and CH_3_COONa (1.76 g, 22.0 mmol) were added to a flat-bottomed flask. Next, 30 mL of MeOH was added, followed by the introduction of compound **9** (1.28 g, 5.2 mmol) into the mixture. The reaction mixture was stirred on a magnetic stirrer heated to 40 °C for 24 h. Then, the same amount of NH_2_OH·HCl and CH_3_COONa was added to the mixture, which was stirred with heating for another 24 h. At the end, the mixture was diluted with H_2_O and extracted with EtOAc (3 × 30 mL). The extract was dried and evaporated. The resulting product was a light cream powder (1.25 g, 97%).

^1^H NMR (400 MHz, CDCl_3_): *δ* 8.84 (br. s, 1H), 8.46 (s, 1H), 7.24 (d, *J* = 7.5 Hz, 1H), 7.19 (t, *J* = 7.8 Hz, 1H), 6.99 (td, *J* = 7.5, 0.5 Hz, 1H), 6.86 (d, *J* = 7.6 Hz, 1H), 4.43 (t, *J* = 8.6 Hz, 1H), 2.99 (dd, *J* = 13.9, 7.8 Hz, 1H) 2.68 (dd, *J* = 14.0, 9.6 Hz, 1H), 1.09 (s, 9H). ^13^C NMR (100 MHz, CDCl_3_): *δ* 180.2, 163.9, 141.2, 129.5, 128.0, 125.3, 122.2, 109.4, 42.1, 37.7, 28.0, 27.2. HRMS-ESI, *m/z*: [M + H]^+^ calculated for C_14_H_19_N_2_O_2_^+^ 247.1441, obtained 247.1454.

*1-(1H-Indol-3-yl)-3,3-dimethyl-butan-2-amine (13)*

Compound **10** (0.74 g, 3.0 mmol) was dissolved in 20 mL of MeOH. A catalytic amount of PtO_2_ was added to the solution. The reaction was carried out in a hydrogen atmosphere (6 bar). Thereafter, the mixture was stirred at room temperature for 48 h. Afterwards, the precipitate that formed was filtered off and dried. Due to its instability, the resulting product was immediately introduced to the next stage of the synthesis. NaBH_4_ (0.57 g, 15.1 mmol) was added to 10 mL of freshly distilled THF, which was in a flat-bottomed conical flask with a stirrer. The flask was placed in an ice bath; its contents were cooled to 0 °C, after which freshly distilled BF_3_·OEt_2_ (2.043 mL, 16.6 mmol) was added in portions. The ice bath was removed, and the reaction mixture was stirred for another 15 min at room temperature. The previously obtained product was added to the solution. The mixture was heated to reflux. After 2 h, the mixture was cooled to room temperature, after which a 10% HCl solution was added to it to a fivefold dilution. The mixture was again heated to reflux and after 2 h was cooled to room temperature. The mixture was then neutralized with Na_2_CO_3_ (aq) and extracted with EtOAc (3 × 30 mL). The extract was dried, evaporated under reduced pressure, and immediately introduced to the next reaction. Product **11** was not isolated or characterized due to low stability.

*1-(2′-Iodobenzoyl)-3-tert-butyl-β-carboline (14)*

2′-Iodoacetophenone (0.5 mmol) and iodine (0.09 g, 0.4 mmol) were added to 2 mL of DMSO, and the resulting solution was heated at 110 °C for 1 h. Afterwards, tryptamine **11** (0.5 mmol) was added to the solution, and this solution was stirred at the same temperature for 3–4 h until the completion of the reaction (monitored by TLC). Then, the reaction mixture was cooled to room temperature followed by the addition of H_2_O (50 mL) and extraction with EtOAc (2 × 25 mL). The extract was washed with 10% Na_2_S_2_O_3_, dried over Na_2_SO_4_, filtered, and evaporated under reduced pressure. The residue was purified by MPLC using benzene as an eluent to give the desired product **12** (yellow solid, 6%).

^1^H NMR (400 MHz, CDCl_3_): *δ* 10.20 (br. s, 1H), 8.20 (s, 1H), 8.18 (dd, *J* = 8.0, 0.5 Hz, 1H), 7.67 (d, *J* = 8.0, 0.8 Hz, 1H), 7.61–7.58 (m, 3H), 7.44 (td, *J* = 7.5, 1.0 Hz, 1H), 7.38–7.31 (m, 2H), 1.35 (s, 9H). ^13^C (100 MHz, CDCl_3_): *δ* 198.2, 158.4, 141.4, 140.8, 135.1, 133.4, 132.6, 132.5, 130.7, 130.2, 129.0, 126.3, 121.7, 121.1, 120.6, 120.4, 114.4, 111.9, 37.5, 30.5. HRMS-ESI, *m/z*: [M + H]^+^ calculated for C_22_H_20_IN_2_O^+^ 455.0615, obtained 455.0624.

*1-(2*′*-Bromobenzoyl)-4-tert-butyl-β-carboline (6)*

A solution of 2′-bromoacetophenone (28 mg, 0.14 mmol) and iodine (28 mg, 0.11 mmol) in DMSO (1 mL) was heated at 110 °C for 1 h. Next, 2-(3-indolyl)-3,3-dimethylbutylamine (30 mg, 0.14 mmol) in DMSO (1 mL) was added to the solution and stirred at 110 °C for 4 h. The reaction mixture was cooled, then poured to water and N_2_S_2_O_3_ was added. The formed precipitate was filtered off, and the filtrate was discarded. The precipitate was washed with ethyl acetate and the solvent was evaporated under reduced pressure. The residue was purified by MPLC using benzene as eluent. The product is a yellow solid (22 mg, 38%).

^1^H NMR (400 MHz, CDCl_3_): *δ* 10.88 (br. s, 1H), 8.63 (s, 1H), 8.47 (d, J = 8.3 Hz, 1H), 7.69 (t, J = 7.5 Hz, 2H), 7.63 (t, J = 7.5 Hz, 1H), 7.53 (dd, J = 7.6, 1.7 Hz, 1H), 7.47 (td, J = 7.5, 1.0 Hz, 1H), 7.40 (m, 2H), 1.75 (s, 9H); ^13^C NMR (100 MHz, CDCl_3_): *δ* 198.2, 145.5, 141.2, 140.7, 137.5, 136.9, 133.8, 133.0, 131.0, 129.5, 128.7, 128.3, 127.4, 126.8, 120.5, 119.9, 119.8, 112.2, 35.2, 29.5; HRMS-ESI, m/z: [M]^+^ calcd. for C_22_H_19_BrN_2_O, 407.3031; found, 407.3027.

*6-tert-Butylfascaplysin (6-TB)*

A solution of β-carboline **12** (0.05 mmol) in 10 mL of acetonitrile was irradiated with UV for 30–90 min. The solution was evaporated, the residue was washed from the starting β-carboline with acetonitrile or benzene, depending on the solubility of the resulting fascaplysin. For re-irradiation, the non-reacted β-carboline solution was evaporated, dissolved in 10 mL of acetonitrile, and irradiated again. The end of the reaction was monitored by TLC. After filtration, the fascaplysin was washed with EtOH, then evaporated under reduced pressure and dried. Then, the product was dissolved in H_2_O, and an aqueous solution of Na_2_CO_3_ was added. The resulting dark green precipitate of the deprotonated form of the product was filtered, washed with water, and washed off with an aqueous solution of HCl. The resulting solution was evaporated and dried. The product is a red solid (93%).

^1^H NMR (400 MHz, CD_3_OD): *δ* 9.00 (s, 1H), 8.53 (d, *J* = 8.0 Hz, 1H), 8.43 (d, *J* = 8.6 Hz, 1H), 8.08 (d, *J* = 6.8 Hz, 1H), 7.96 (t, *J* = 7.6 Hz, 1H), 7.85 (t, *J* = 7.6 Hz, 1H), 7.77–7.71 (m, 2H), 7.48 (t, *J* = 7.5 Hz, 1H), 1.95 (s, 9H). ^13^C NMR (100 MHz, MeOH-d4): *δ* 182.9, 149.1, 148.8, 141.5, 136.1, 134.7, 131.5, 130.5, 125.5. 125.3, 124.8, 124.4, 124.2, 122.9, 119.6, 119.2, 113.2, 112.0, 36.6, 30.4. HRMS-ESI, *m/z*: [M]^+^ calculated for C_22_H_19_N_2_O^+^ 327.1492, obtained 327.1486.

*7-tert-Butylfascaplysin (7-TB)*

1-(2′-Bromobenzoyl)-4-*tert*-butyl-β-carboline (17 mg, 0.04 mmol) was heated at 220 °C for 30 min. The product was washed with EtOAc until coloring ceased. The red precipitate was dissolved in hot water and filtered. Sodium carbonate was added to the solution, and a green precipitate formed. The precipitate was filtered, dissolved with acidified EtOH, the substance went to solution, and the color became red. The solvent was evaporated under reduced pressure. The product is a red solid (14 mg, 93%).

^1^H NMR (400 MHz, CD_3_OD): δ 9.00 (s, 1H), 8.63 (d, J = 8.6 Hz, 1H), 8.50 (d, J = 8.1 Hz, 1H), 8.04 (d, J = 7.3 Hz, 1H), 7.97 (t, J = 7.6 Hz, 1H), 7.88 (m, 2H), 7.75 (t, J = 7.5, 1H), 7.59 (m, 1H), 1.87 (s, 9H); ^13^C NMR (100 MHz, CD_3_OD): δ 181.8, 147.7, 147.4, 147.1, 138.0, 136.7, 133.1, 132.4, 131.3, 128.6, 125.3, 124.3, 122.8, 122.7, 120.7, 118.6, 115.3, 113.5, 35.6, 27.9; HRMS-ESI, m/z: [M]^+^ calcd. for C_22_H_19_N_2_O, 327.3985; found, 327.3981.

### Biology

#### Reagents and antibodies

MTT (3-(4,5-dimethylthiazol-2-yl)-2,5-diphenyltetrazolium bromide) was purchased from Sigma (Taufkirchen, Germany), annexin-V-FITC from BD Bioscience (San Jose, CA, USA); tariquidar (p-glycoprotein inhibitor) from MedChemExpress (Monmouth Junction, NJ, USA); calcein-AM from BIOZOL (Eching, Germany); VE-822 (ATR inhibitor), LY2603618 (Chk1 inhibitor), KU-60019 (ATM inhibitor), and PV1019 (Chk2 inhibitor) from LC Laboratories (Woburn, MA, USA); Anisomycin—from NeoCorp (Weilheim, Germany). Docetaxel, and doxorubicin—from a Pharmacy of the University Hospital Hamburg-Eppendorf (Hamburg, Germany). Primary and secondary antibodies used are listed in Supplementary Table S1.

#### Cell lines and culture conditions

The human prostate cancer LNCaP, 22Rv1, PC-3, and DU145 cell lines and human prostate non-cancer PNT2 cells were purchased from ATCC (Manassas, VA, USA). Human non-cancer human embryonic kidney HEK 293 T cells and human fibroblast MRC-9 cells were purchased from ECACC (Salisbury, UK). The human prostate cancer docetaxel-resistant PC3-DR cell line was generated by long-term incubation with docetaxel as previously described^43^ and were kindly provided by Prof. Z. Culig, Innsbruck Medical University, Austria. The cells lines were recently authenticated by Multiplexion GmbH (Heidelberg, Germany). Cells were cultured as previously described for maximum 3 months and were regularly examined for mycoplasma infection.

#### Trypan blue exclusion assay

The trypan blue exclusion assay was performed to access membrane integrity. Cells were plated in 6-well plates (2 × 10^5^ cells/well) per well in 2 mL/well, incubated overnight, and treated with drugs at indicated concentrations in fresh medium. Cells were incubated for 48 h, harvested using trypsinization, stained with trypan blue dye, and trypan blue-negative alive cells were conducted using the automatic Beckman Coulter Vi-CELL (Beckman Coulter, Krefeld, Germany).

#### DNA intercalation assay

The DNA-intercalating activity of the compounds was assessed using thiazole orange (TO) displacement method from double-stranded DNA as reported previously^37^. In brief, for this analysis we used mixture containing 1 µM of double-stranded calf thymus DNA and 2 µM of thiazole orange (TO) in water. The solutions of tested compounds in DMSO were added to the mixture ensuring that the maximum DMSO concentration in the samples do not exceed 0.02%. Propidium iodide (PI) was used as a positive control. The samples were incubated for 7 min at room temperature, and the TO fluorescence was measured using a TECAN Spark multimodal plate reader (Tecan Group Ltd., Männedorf, Switzerland). The effective concentration (EC_50_) was calculated using GraphPad Prism software v.9.1.1 (GraphPad Software, San Diego, CA, USA).

#### p-gp activity assay

The assessment of p-glycoprotein (p-gp) activity was done using calcein-AM as previously reported^59^. Cells were plated in black 96-well plates with clear bottoms at a density of 10 × 10^3^ cells/well in 100 µL/well, incubated overnight, and the medium was replaced with 50 µL of a drug solution in PBS. Following 30 min incubation, 50 µL of calcein-AM solution in PBS was added to each well to achieve a final concentration of 1 µM. The plates were then incubated for an additional 15 min, and the green fluorescence was immediately measured using an Infinite F200PRO reader (TECAN). The viability of cells treated in the same conditions with the drug was accessed using the MTS assay as previosuly reported^59^.

#### MTT assay

Cell viability was assessed using the MTT assay. Cells were plated in 96-well plates (6 × 10^3^ cells/well in 100 μL/well, incubated overnight, and the medium was replaced with fresh drug-containing medium containing and incubated for 48 h unless otherwise indicated. Then MTT reagent was added to each well and the plates were then incubated for another 2–4 h. The media was removed, and the plates were dried overnight, and 50 μl/well of DMSO was added to dissolve the formazan crystals. The absorbance of these solutions was measured using an Infinite F200PRO reader (TECAN, Männedorf, Switzerland). The inhibition concentration (IC_50_) was calculated using GraphPad Prism software v.9.1.1 (GraphPad Software, San Diego, CA, USA).

#### Functional kinome profiling

Functional kinome profiling was performed as previously reported^60^. In brief, the PamStation^®^12 system (PamGene International, ´s-Hertogenbosch, The Netherlands) and STK-PamChip^®^ array designed for the analysis of serine-/threonine kinase activities were used. Each array comprises 140 distinct peptide phospho-sites that serve as analogs for the substrates of corresponding serine-/threonine kinases. The whole cell lysates were prepared using M-PER Mammalian Extraction Buffer (Pierce, Waltham, Massachusetts, USA), supplemented with protease and phosphatase inhibitor cocktails, following 2 h incubation of the cells with 7-TB. A mixture of total protein extract and ATP was prepared and applied in the array, and the phosphorylation of specific peptide sequences was then detected using primary anti-phospho-Ser/Thr antibodies, secondary immunoglobulin-FITC antibody (PamGene International). The signals were recorded using a CCD camera and Evolve software v. 1.0 (PamGene International), and the quality of the signals was monitored. The final signal intensities were log2-transformed and analyzed using the BioNavigator software v. 6.0 (BN6, PamGene International).

#### Western blotting

Western blotting was performed as discribed previously^61^. In brief, 10^6^ cells were seeded per dish in Petri dishes, incubated overnight and treated with the drug for 48 h unless otherwise stated. Cells were harvested by scratching, washed and lyzed using RIPA buffer containing protease and phosphotase inhibitors. Proteins were saparated using SDS-PAGE and Mini-PROTEAN^®^ TGX Stain-FreeTM gels (Bio-Rad, Hercules, CA, USA), transferred onto PVDF membranes and visualized using appropriate primary and secondary antibodies. The signals were detected using ECL chemiluminescence system (Thermo Scientific, Rockford, IL, USA). The antibodies used are listed in Supplementary Table S1. The original images obtained were cropped and the figures were composed using MS PowerPoint software v. 2310, build 16,924,20,150 (Microsoft Inc., Redmond, WA, USA). The original uncroped images are represented in Supplementary Figure S2.

#### Colony formation assay

Colony formation assay was conduced as previously described^62^. Briefly, 22Rv1, DU145 or PC-3 cells were seeded in 6-well plates with a denisty of200 cells/well in 2 mL/well, and incubated overnight. On the next day the tested drugs were added to the cells at the indicated concentrations an the cells were incubated for a period of 14 days to allow colony formation. The resulting colonies were fixed using 70% ethanol, stained with 0.1% crystal violet, and counted by eye.

#### Flow cytometry analysis

22Rv1 cells were plated and as described above for Trypan blue exclusion assay and incubated overnight. Then, the cells were pre-treated with 100 µM z-VAD(OMe)-fmk for 1 h and then treated with the tested drugs for another 48 h. Cells were collected using trypsination, stained with annexin-V-FITC and propidium iodide, and analyzed using a FACS Calibur instrument (BD Bioscience, San Jose, CA, USA) as previously reported^61^. The generated data were analyzed using the Cell Quest Pro software version 5.2.1 (BD Bioscience).

#### Immunofluorescence

Cells were placed on coverslips and treated for indicated times. The following concentrations of 6-TB and 7-TB were used, respectively: 0.5 μM and 0.1 μM for PC3 cells, 0.25 μM and 0.05 μM for 22Rv1 cells, and 1 μM and 0.2 μM for DU145 cells. After the treatment, cells were washed once with cold PBS, then fixed using 4% paraformaldehyde/PBS for 10 min. Following fixation, the cells were permeabilized using 0.2% Triton X-100/PBS on ice for 5 min. The slides were incubated with primary anti-phospho-S139-H2AX and anti-53BP1 antibodies for 1 h at room temperature. The cells were washed three times with cold PBS and incubated for 1 h with secondary anti-mouse Alexa-fluor594 and anti-rabbit Alexa-fluor488 antibodies. Nuclei were additionally stained with DAPI (10 ng/mL) and the slides were mounted using Vectashield mounting medium (Vector Laboratories, Newark, CA, USA). Fluorescence microscopy was performed using a Zeiss AxioObserver.Z1 microscope, equipped with objectives of × 20 (resolution 0.44 µm) and Plan Apo 63/1.4 Oil DICII (resolution 0.24 µm), and filters including Zeiss 43, Zeiss 38, and Zeiss 49 (Carl Zeiss, Göttingen, Germany). Z-stacks of semi-confocal images were captured using the Zeiss Apotome, Zeiss AxioCam MRm, and Zeiss AxioVision Software (Carl Zeiss, Göttingen, Germany). The quantification of DSBs was performed using ImageJ (version 1.48; https://imagej.net/ij/), applying DAPI-based image masks, and the results were normalized to values per individual nucleus. The antibody used are listed in the Supplementary Table S1.

#### Fiber assay

Cells were seeded and treated with investigated drugs for 24 h as described above for the Immunofluorescence assay. The cells were washed with PBS and sequentially pulse-labeled with CldU (25 μM) and then with IdU (250 μM), for 20 min each. Following labeling the cells were harvested and DNA fibers were spread and stained according to a previously described method^63^. The stained DNA fibers were then analyzed using an Axioplan 2 fluorescence microscope (Zeiss, Oberkochen, Germany). Analysis of the CldU and IdU tracks on these fibers was conducted using ImageJ software (version 1.48; https://imagej.net/ij/). The minimal number of replication forks analyzed per sample was 300.

#### Data and statistical analysis

Statistical analyses were conducted using GraphPad Prism software version 7.05 (GraphPad Prism software Inc., La Jolla, CA, USA). The data are expressed as mean ± standard deviation (SD). For comparing two groups, the unpaired Student’s t-test was used, while for comparisons among multiple groups, one-way ANOVA followed by Dunnett’s post-hoc tests was applied. Specifically, all experiments were carried out in triplicate (*n* = 3, representing biological replicates) unless otherwise stated. The difference between the groups was considered as statistically significant if *p* < 0.05.

### Supplementary Information

Supplementary Information.

## Data Availability

The original data are available in the Supplementary as well as from the corresponding author on request.
