# Supplementary material for: Synthesis and new DNA targeting activity of 6- and 7-tert-butylfascaplysins
Source: Sci Rep. 2024 May 23;14:11788. doi: 10.1038/s41598-024-62358-8 (PMC11116464; doi:10.1038/s41598-024-62358-8)

Supplementary information

Synthesis and new DNA targeting activity of 6- and 7-*tert*-butylfascaplysins

Sergey A. Dyshlovoy[1,*], Wael Y. Mansour[2,3], Natalia A. Ramm[4], Jessica Hauschild[1], Maxim E. Zhidkov[4], Malte Kriegs[2,5], Alexandra Zielinski[2], Konstantin Hoffer[2,5], Tobias Busenbender[1], Ksenia A. Glumakova[6], Pavel V. Spirin[6,7], Vladimir S. Prassolov[6,7], Derya Tilki[8,9,10], Markus Graefen[10], Carsten Bokemeyer[1], Gunhild von Amsberg[1,10]

1 Laboratory of Experimental Oncology, Department of Oncology, Hematology and Bone Marrow Transplantation with Section Pneumology, Hubertus Wald-Tumorzentrum, University Medical Center Hamburg-Eppendorf, Martinistrasse 52, 20246 Hamburg, Germany; s.dyshlovoy@uke.de (S.A.D.), j.hauschild@uke.de (J.H.), t.busenbender@uke.de (T.B.), c.bokemeyer@uke.de (C.B.), g.von-amsberg@uke.de (G.v.A.)

2 Department of Radiotherapy & Radiation Oncology, Hubertus Wald Tumorzentrum – University Cancer Center Hamburg (UCCH), University Medical Center Hamburg-Eppendorf, Martinistrasse 52, 20246 Hamburg, Germany; w.mansour@uke.de (W.M.); m.kriegs@uke.de (M.Kr.); a.zielinski@uke.de (A.Z.); k.hoffer@uke.de (K.H.)

3 Mildred Scheel Cancer Career Center HaTriCS4, University Medical Center Hamburg-Eppendorf, Martinistrasse 52, 20246 Hamburg, Germany

4 Department of Chemistry and Materials, Institute of High Technologies and Advanced Materials, FEFU Campus, Far Eastern Federal University, Ajax Bay 10, Russky Island, 690922 Vladivostok, Russian Federation; zhidkov.me@dvfu.ru (M.Zh.), rammnataa@gmail.com (N.A.R.)

5 UCCH Kinomics Core Facility, Hubertus Wald Tumorzentrum – University Cancer Center Hamburg (UCCH), University Medical Center Hamburg-Eppendorf, Martinistrasse 52, 20251 Hamburg, Germany

6 Department of Cancer Cell Biology, Engelhardt Institute of Molecular Biology, Russian Academy of Sciences, Vavilova 32, 119991 Moscow, Russian Federation; kglumakova@mail.ru (K.A.G.), spirin.pvl@gmail.com (P.V.S.), prassolov45@mail.ru (V.S.P.)

7 Center for Precision Genome Editing and Genetic Technologies for Biomedicine, Engelhardt Institute of Molecular Biology, Russian Academy of Sciences, Vavilova 32, 119991 Moscow, Russian Federation

8 Department of Urology, University Medical Center Hamburg-Eppendorf, Martinistrasse 52, 20246 Hamburg, Germany

9 Department of Urology, Koc University Hospital, 34010 Istanbul, Turkey

10 Martini-Klinik, Prostate Cancer Center, University Medical Center Hamburg-Eppendorf, Martinistrasse 52, 20246Hamburg, Germany; graefen@martini-klinik.de (M.G.), d.tilki@uke.de (D.T.)

* Correspondence to: Dr. Sergey A. Dyshlovoy, Laboratory of Experimental Oncology, Department of Oncology, Hematology and Bone Marrow Transplantation with Section Pneumology, University Medical Center Hamburg-Eppendorf, Martinistrasse 52, 20251 Hamburg, Germany; e-mail: s.dyshlovoy@uke.de

Contents

Figure S1. Spectra data ………………………………………………………………………. 2-21

Table S1. Primary and secondary antibody used……………………………………..22

Figure S2. Original images used for Figure 5A……………..…………………..…………………..……….23-26

Figure S1. Spectra Data.

^{1}H NMR spectra of 1-(1*H*-indol-3-yl)-2,2-dimethylpropan-1-ol (2).

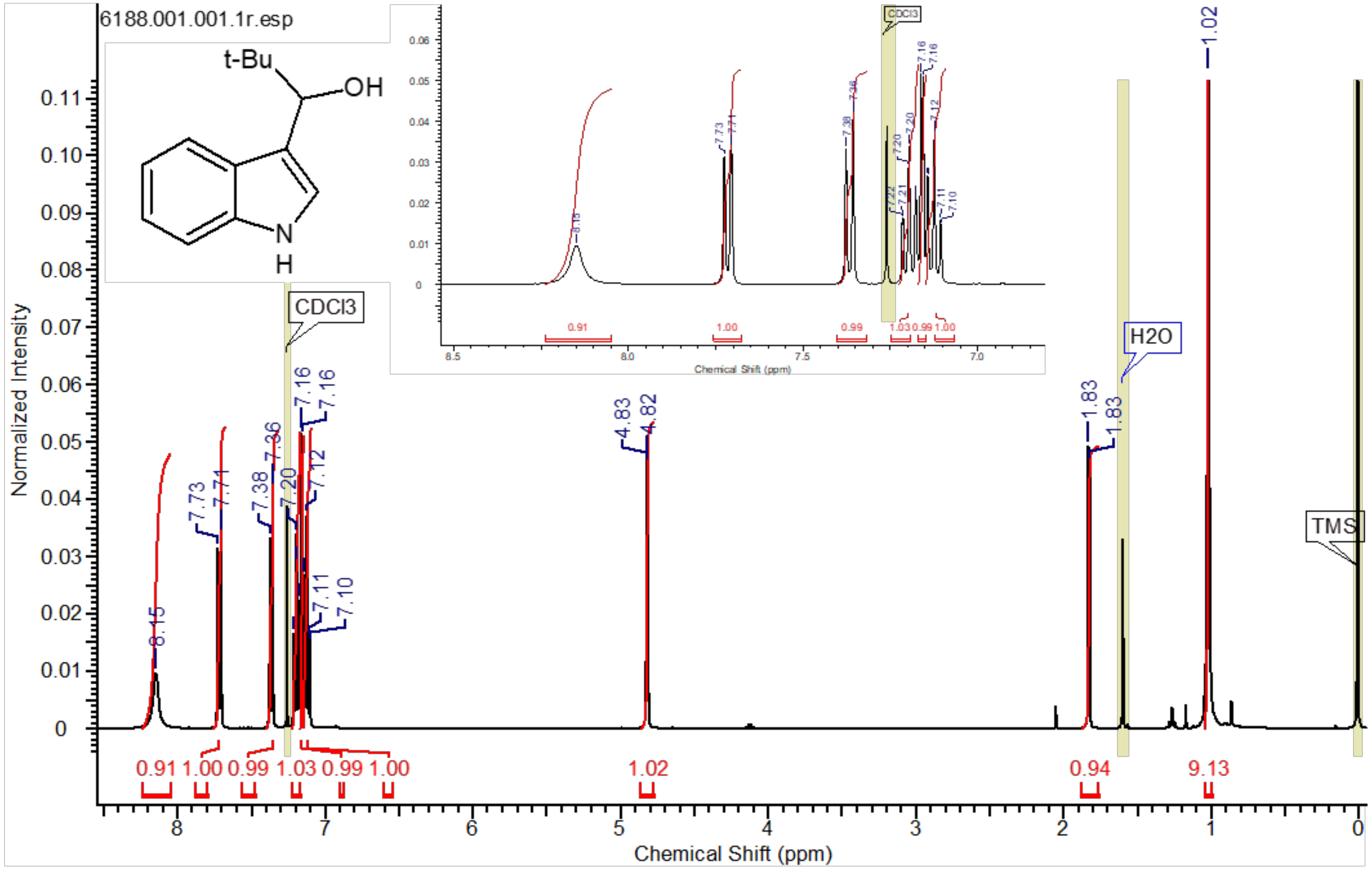

Figure S1. Spectra Data (contunuation)

^{13}C NMR spectra of 1-(1*H*-indol-3-yl)-2,2-dimethylpropan-1-ol (2).

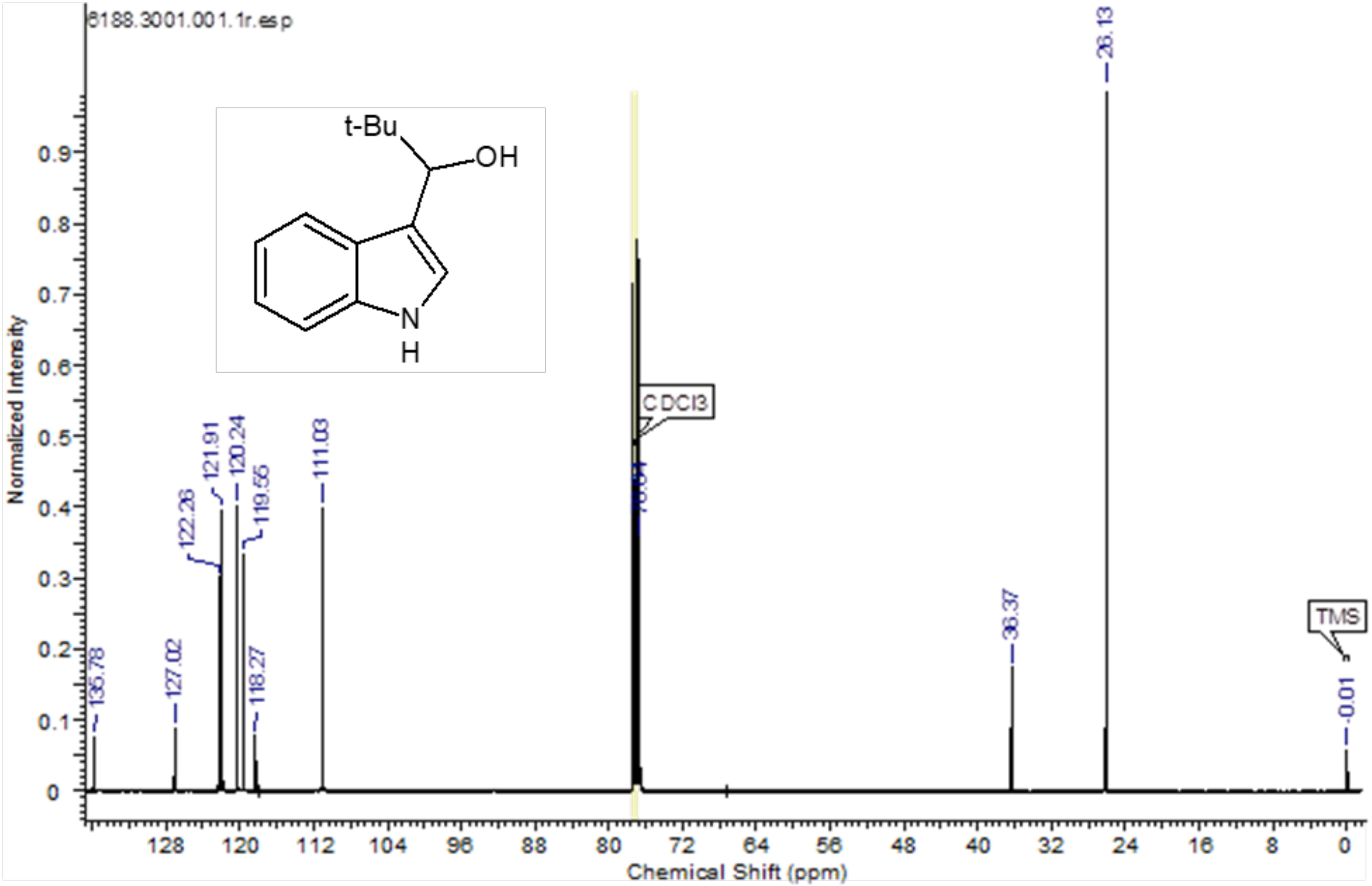

Figure S1. Spectra Data (contunuation)

^{1}H NMR spectra of 2-(1*H*-indol-3-yl)-3,3-dimethylbutanenitrile (3).

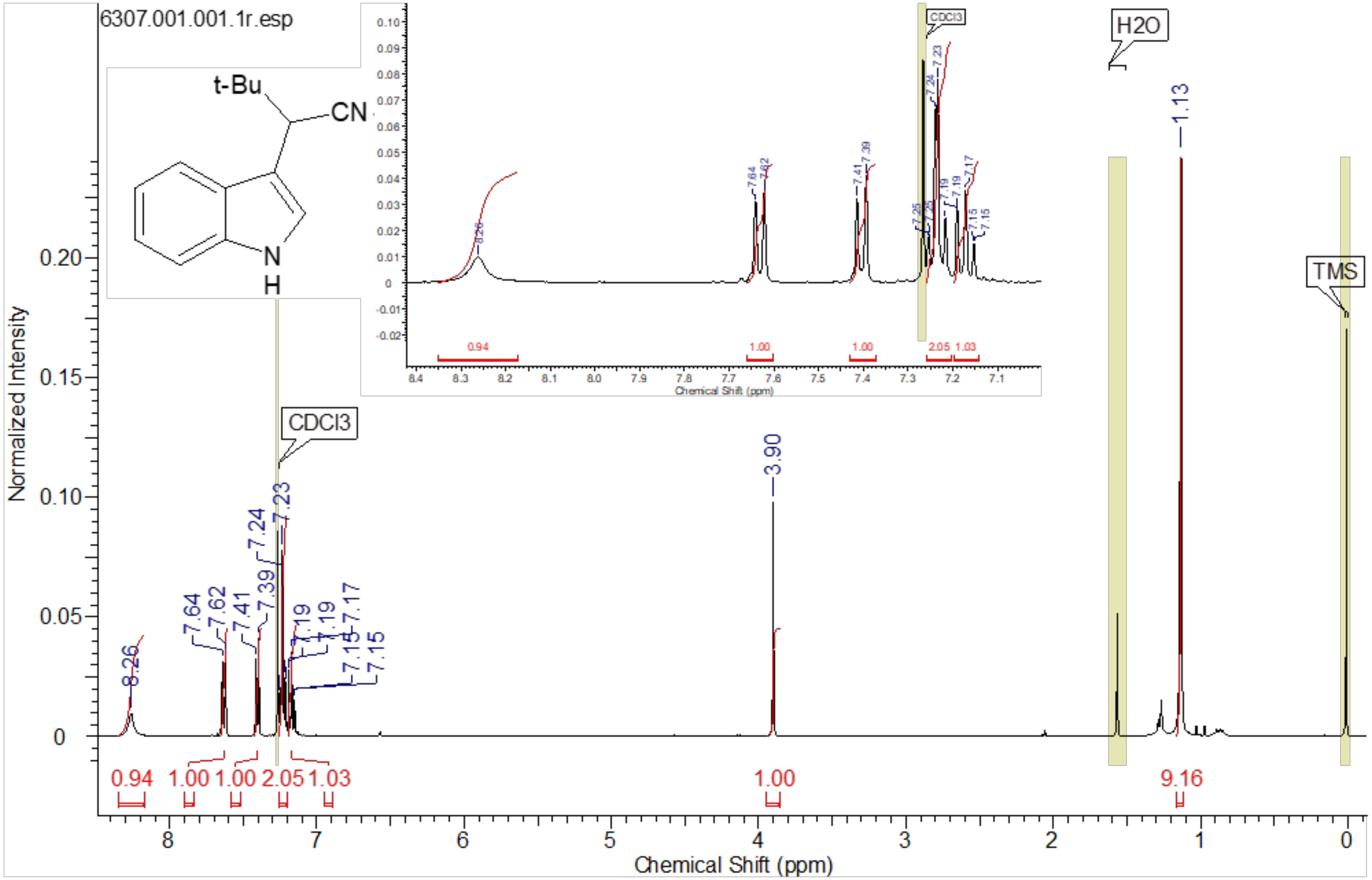

Figure S1. Spectra Data (contunuation)

^{13}C NMR spectra of 2-(1*H*-indol-3-yl)-3,3-dimethylbutanenitrile (3).

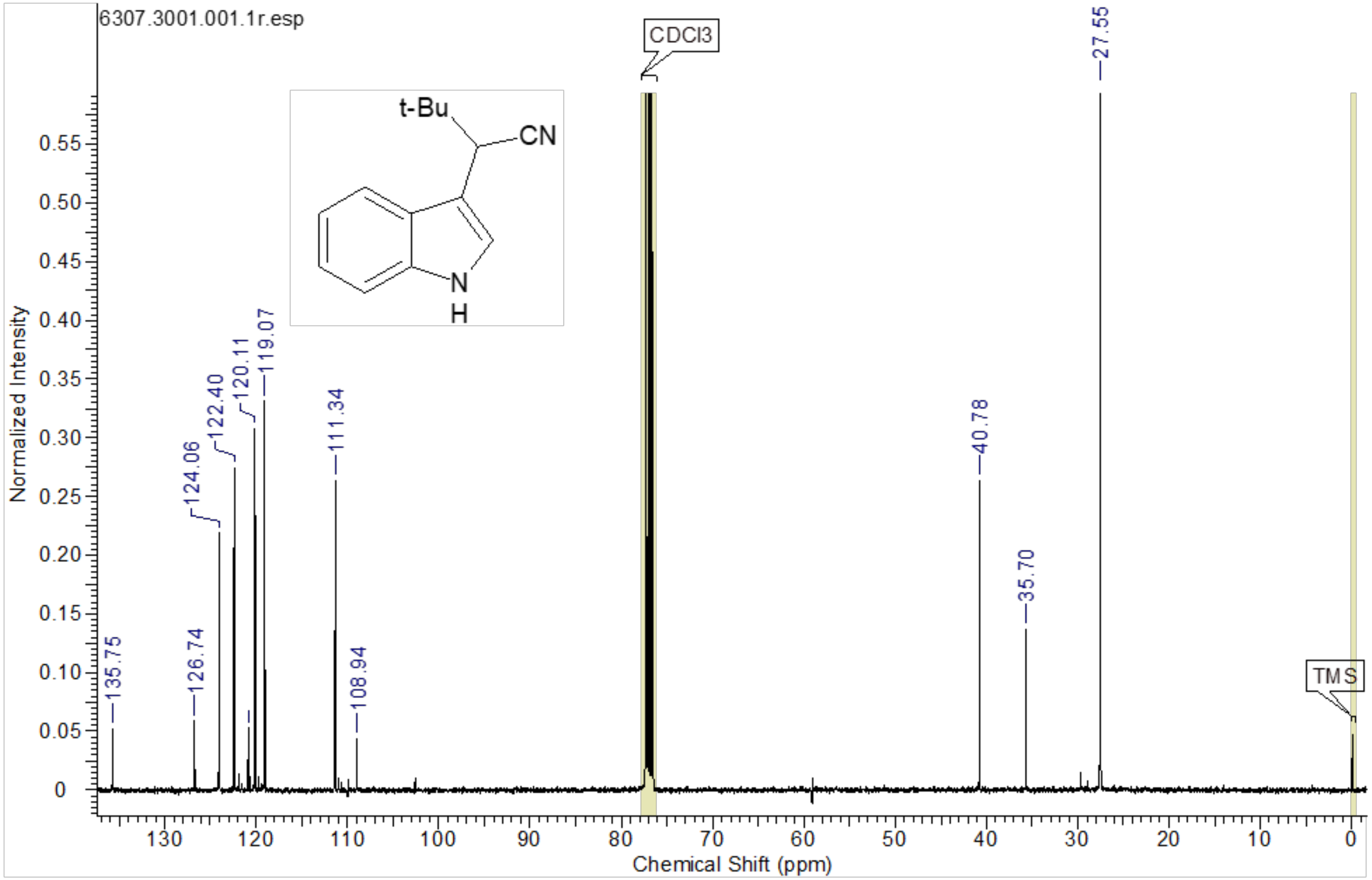

Figure S1. Spectra Data (contunuation)

^{1}H NMR spectra of 3-(3,3-dimethyl-2-oxobutyl)-3-hydroxyindolin-2-one (7)

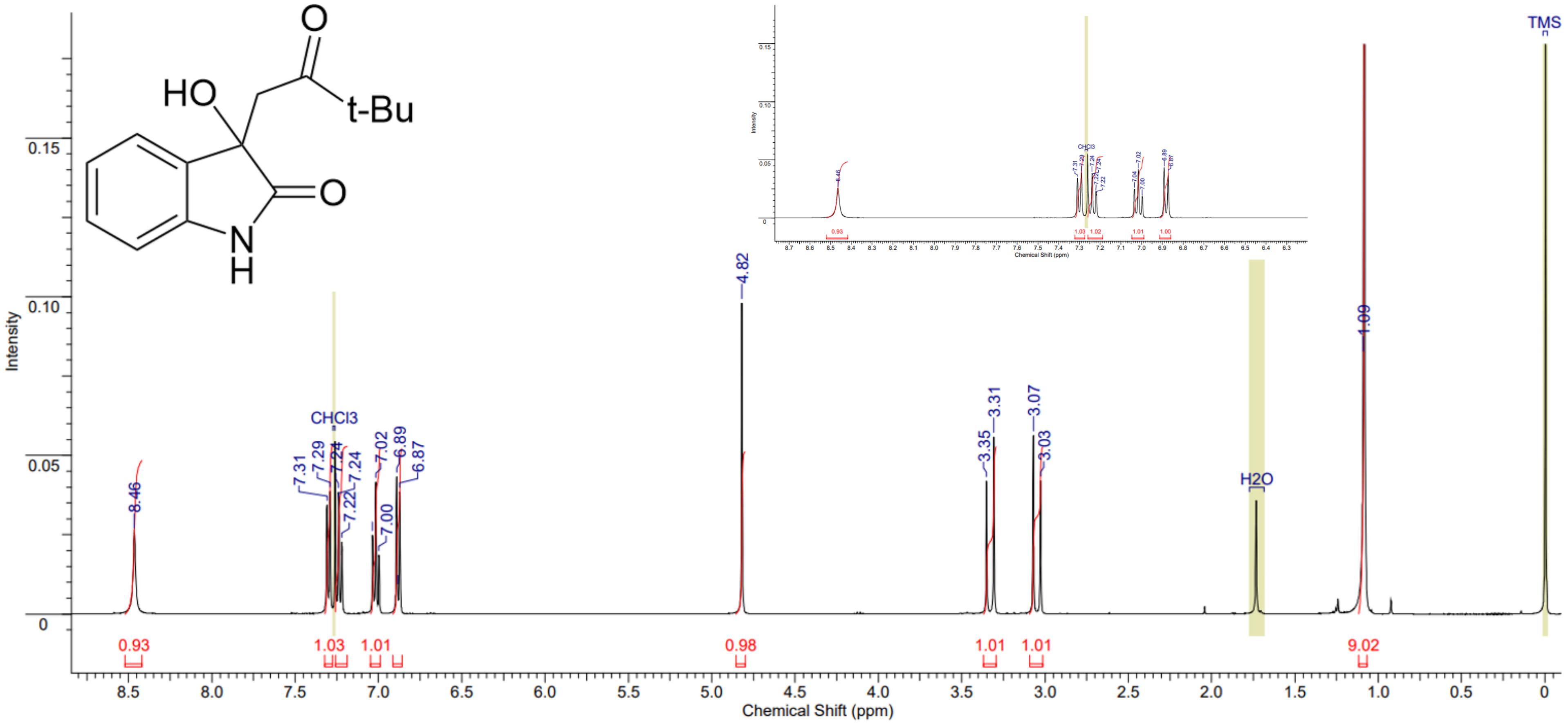

Figure S1. Spectra Data (contunuation)

^{13}C NMR spectra of 3-(3,3-dimethyl-2-oxobutyl)-3-hydroxyindolin-2-one (7)

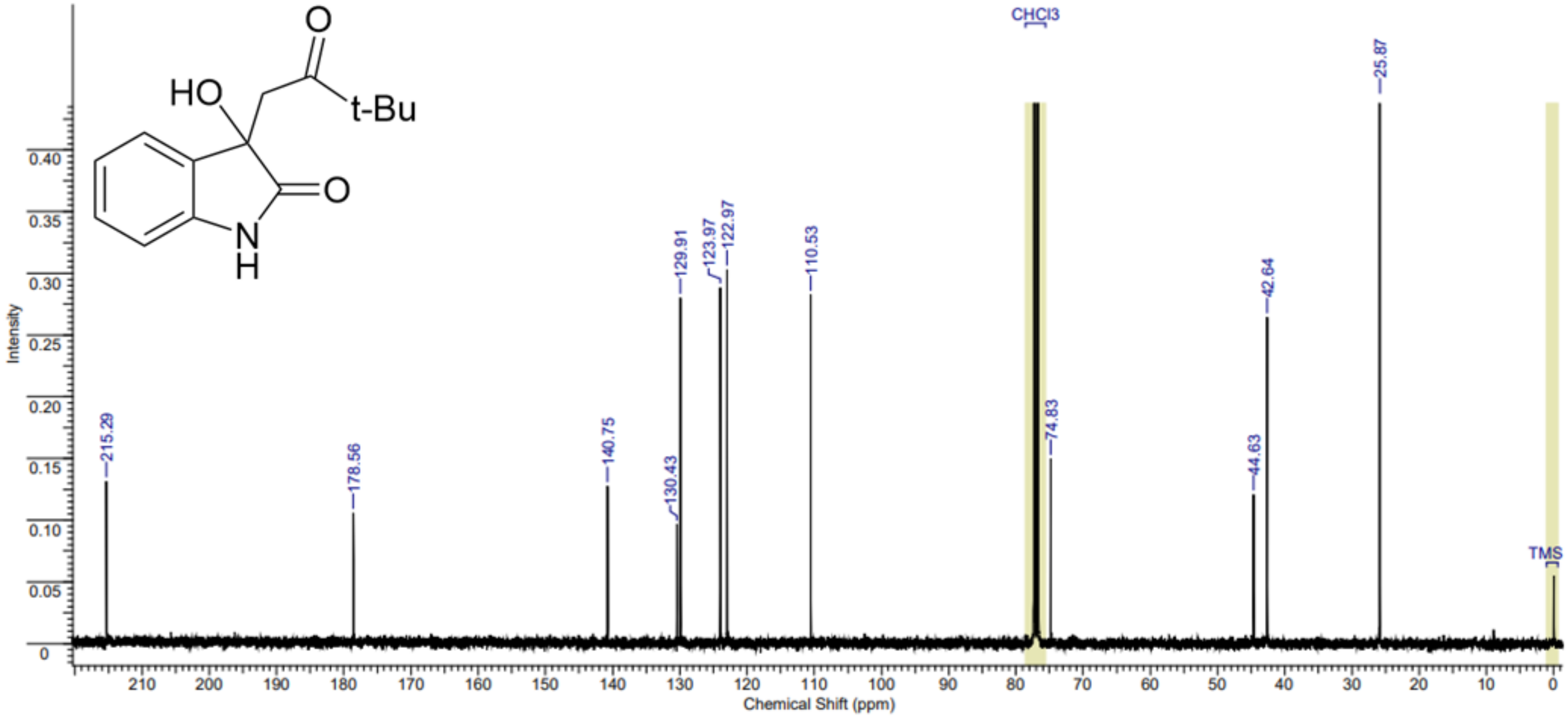

Figure S1. Spectra Data (contunuation)

^{1}H NMR spectra of 3-(3,3-dimethyl-2-oxobuten-4-yl)-indolyn-2-one (8)

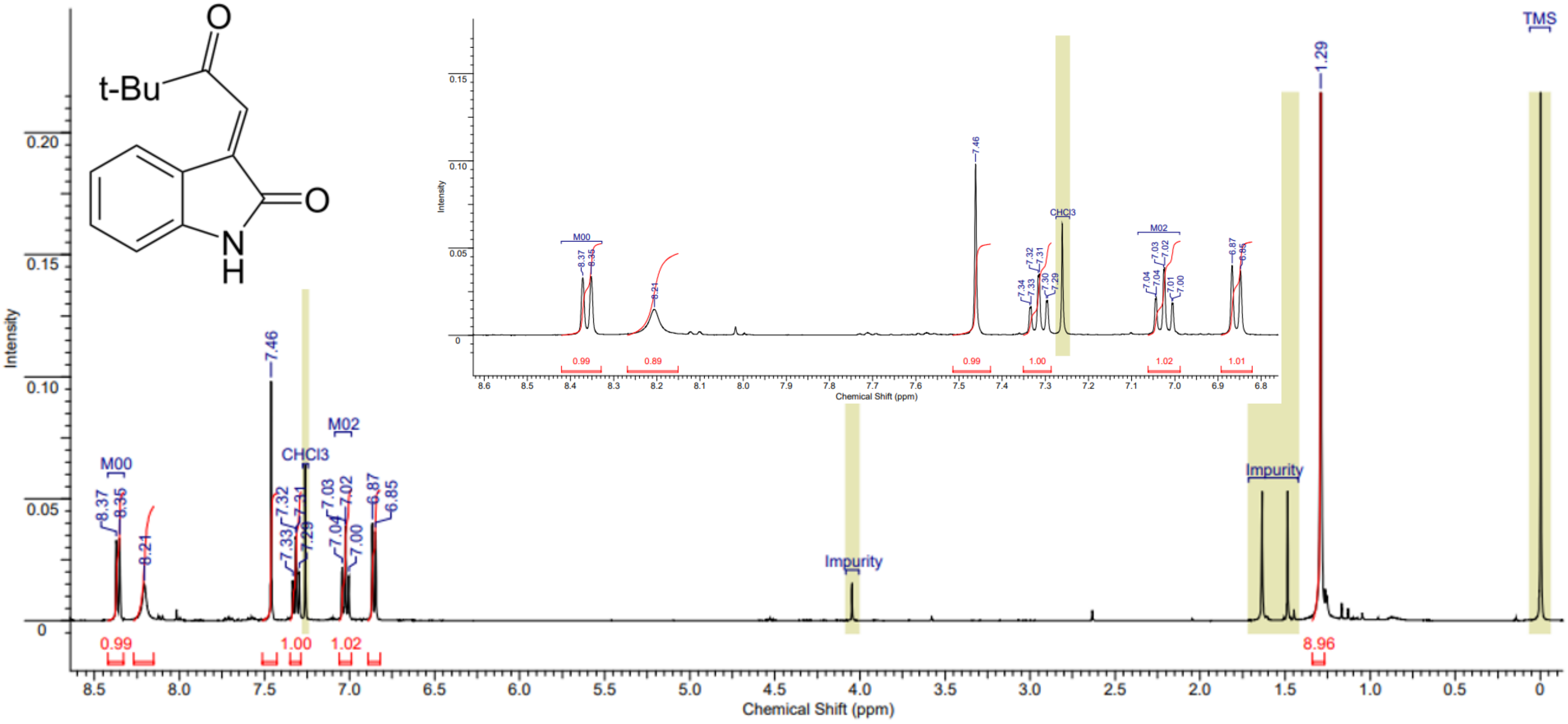

Figure S1. Spectra Data (contunuation)

^{13}C NMR spectra of 3-(3,3-dimethyl-2-oxobuten-4-yl)-indolyn-2-one (8)

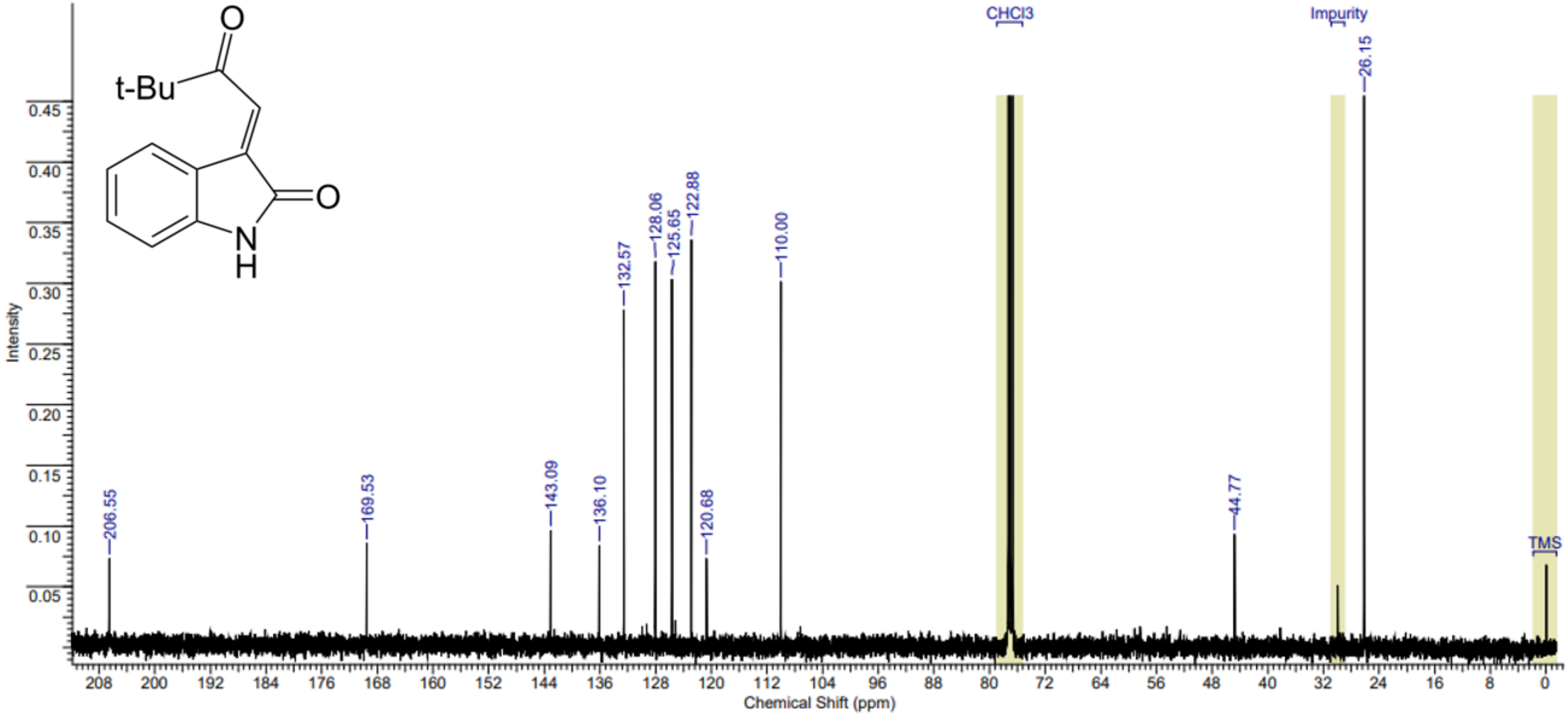

Figure S1. Spectra Data (contunuation)

^{1}H NMR spectra of 3-(3,3-dimethyl-2-oxobutyl)-indolyn-2-one (9)

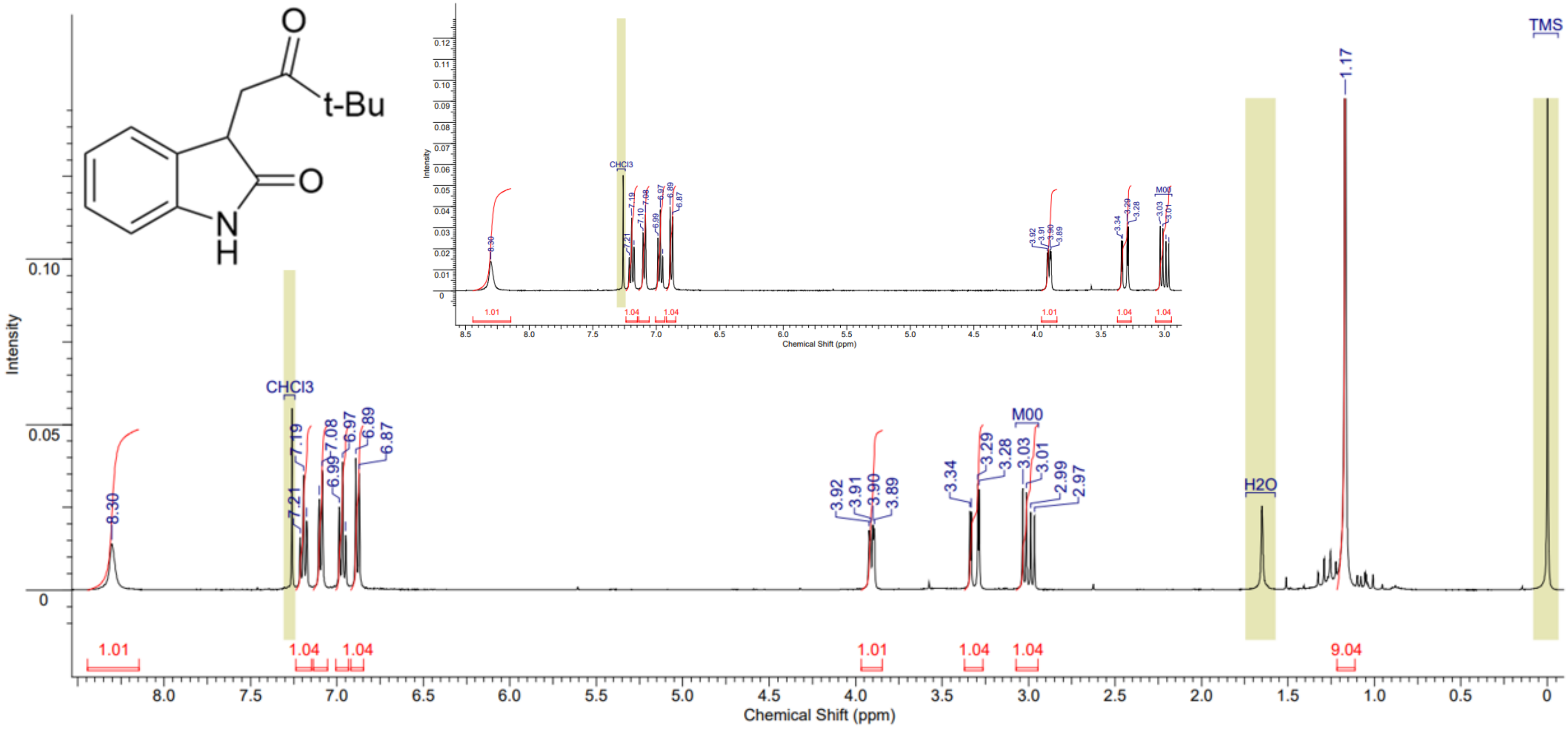

Figure S1. Spectra Data (contunuation)

^{13}C NMR spectra of 3-(3,3-dimethyl-2-oxobutyl)-indolyn-2-one (9)

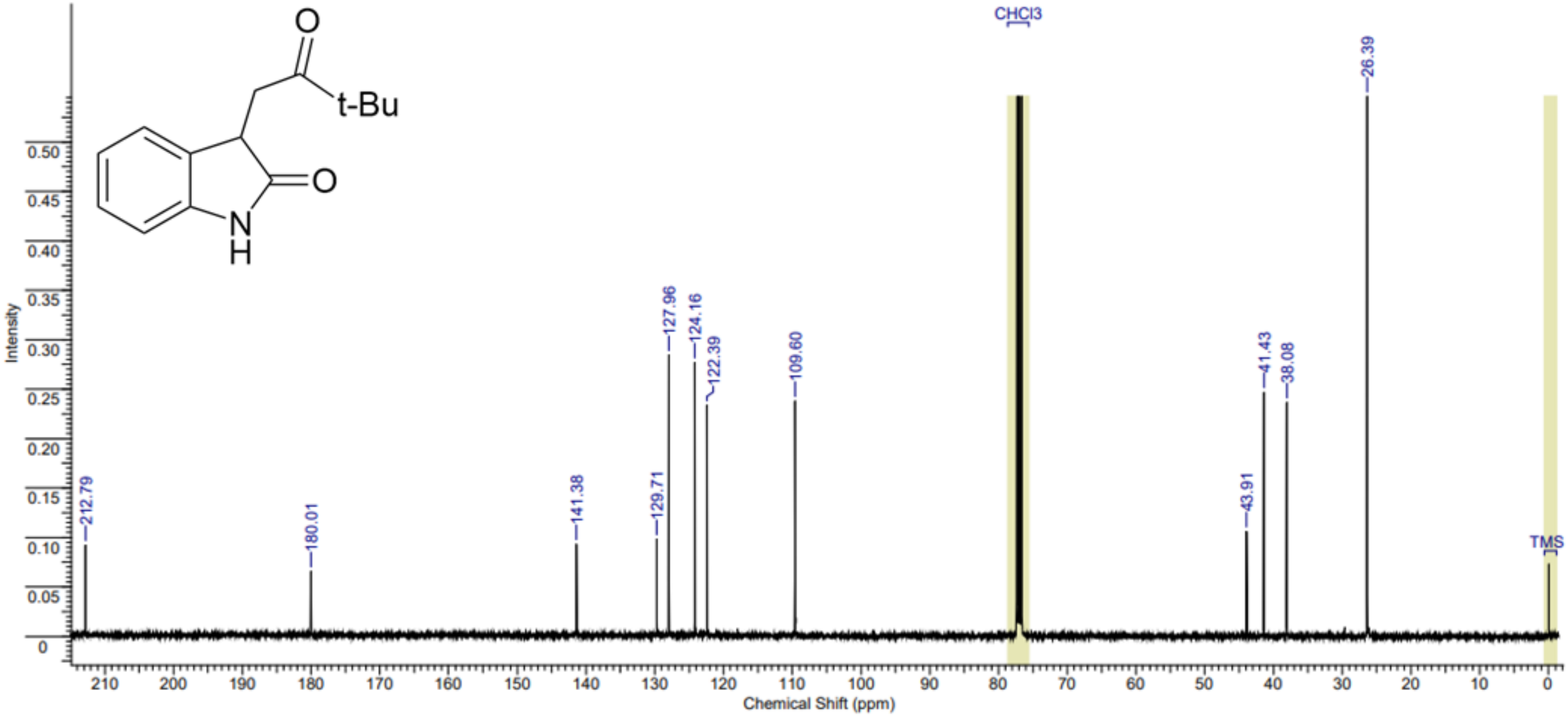

Figure S1. Spectra Data (contunuation)

^{1}H NMR spectra of 3-(2-(hydroxyamino)-3,3-dimethylbutyl)-indolyn-2-one (10)

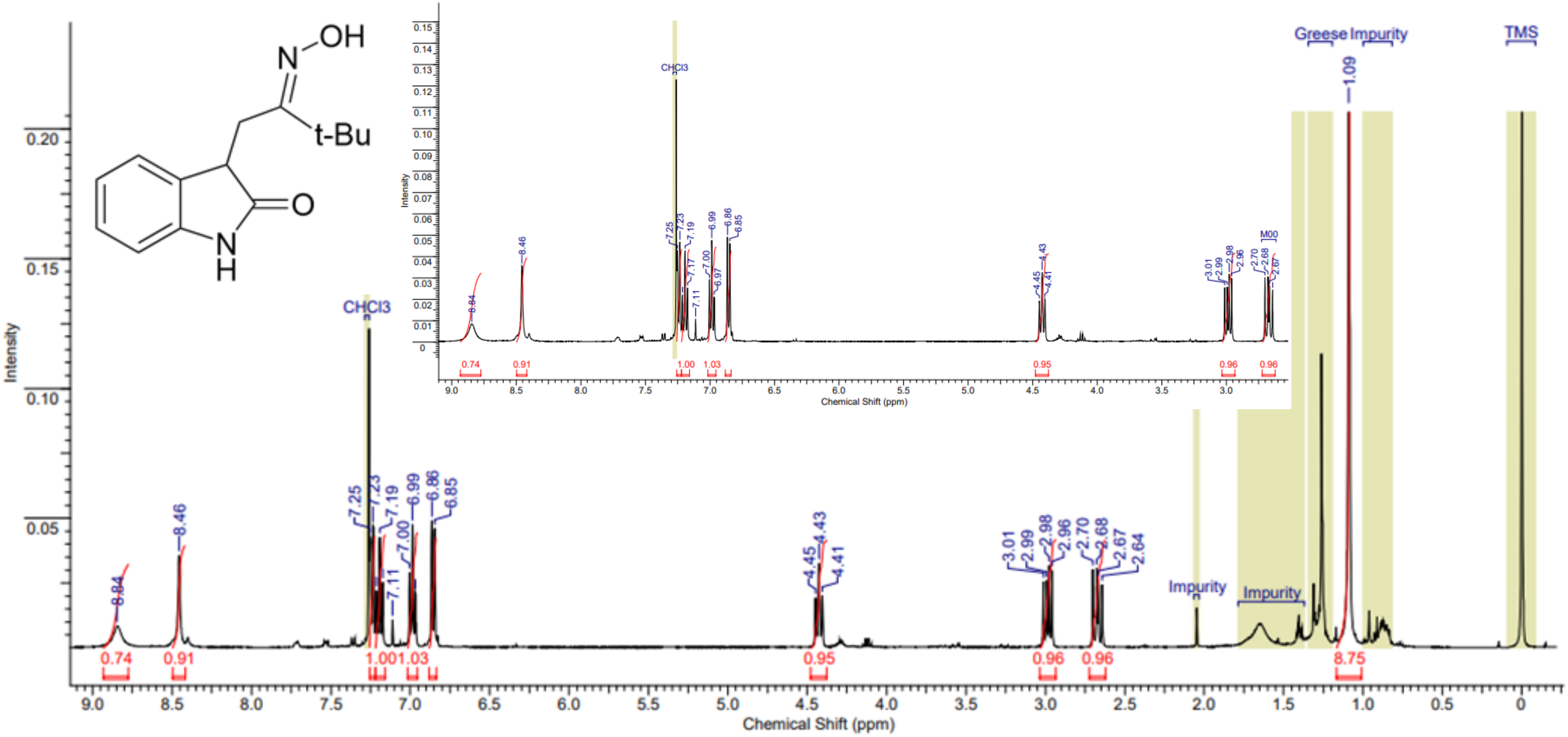

Figure S1. Spectra Data (contunuation)

^{13}C NMR spectra of 3-(2-(hydroxyamino)-3,3-dimethylbutyl)-indolyn-2-one (10)

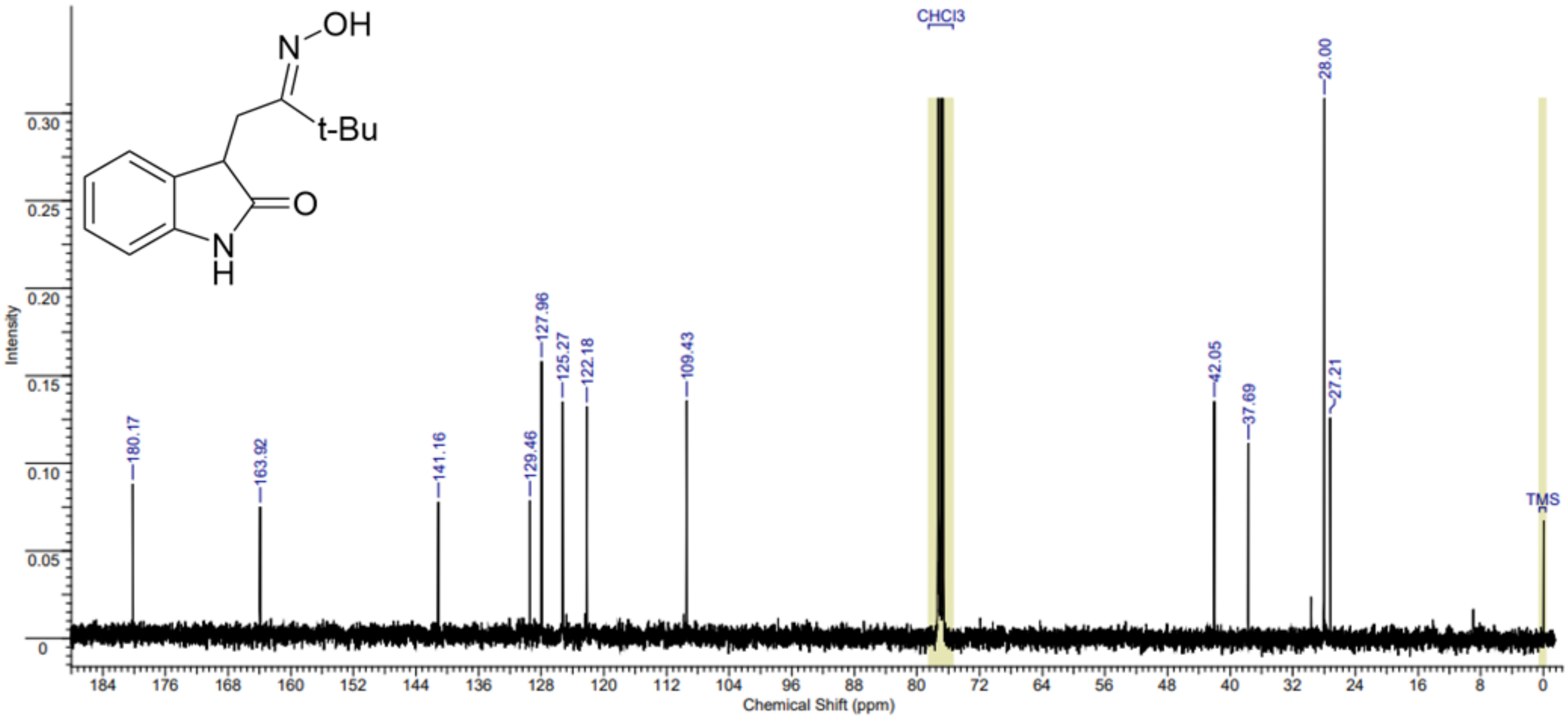

Figure S1. Spectra Data (contunuation)

^{1}H NMR spectra of 1-(2'-iodobenzoyl)-3-*tert*-butyl-β-carboline (12)

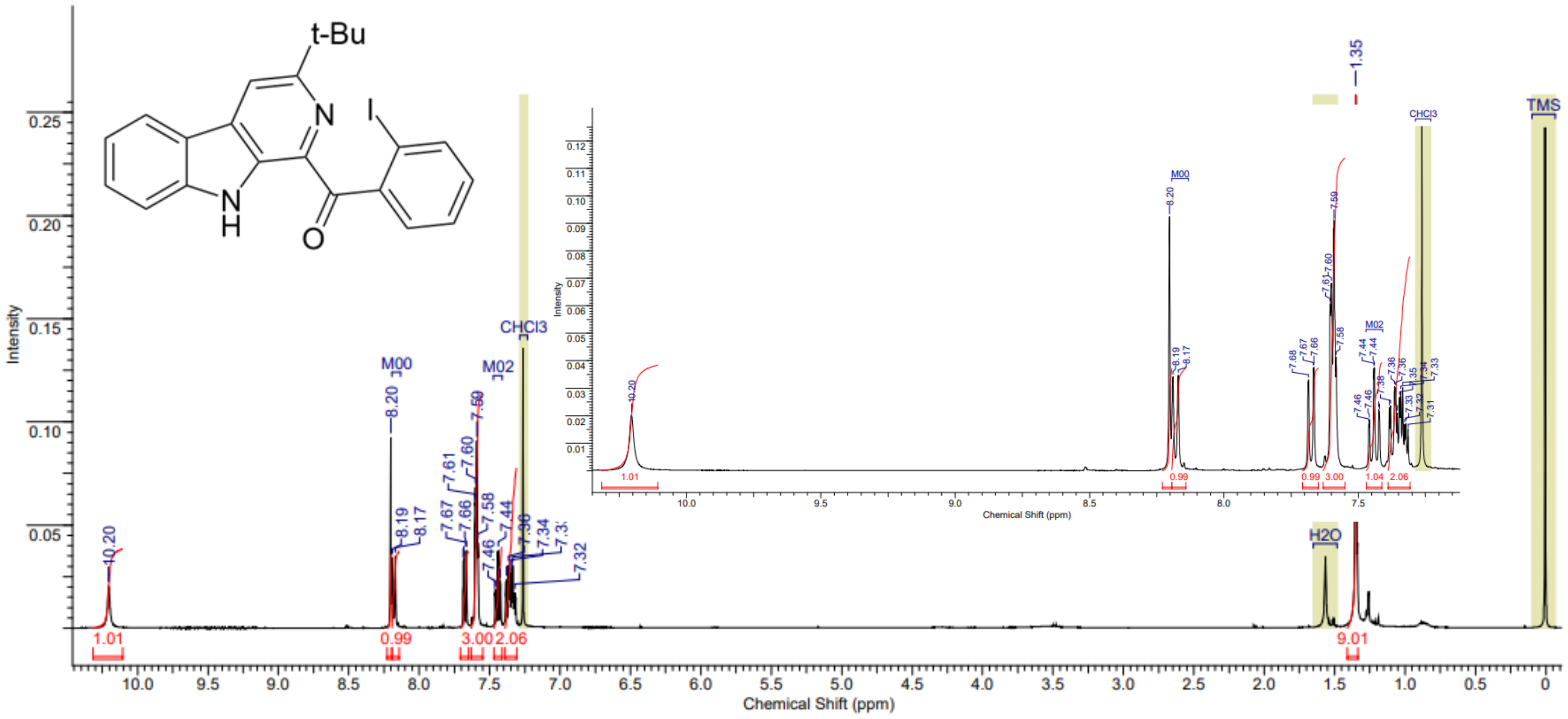

Figure S1. Spectra Data (contunuation)

^{13}C NMR spectra of 1-(2'-iodobenzoyl)-3-*tert*-butyl-β-carboline (12)

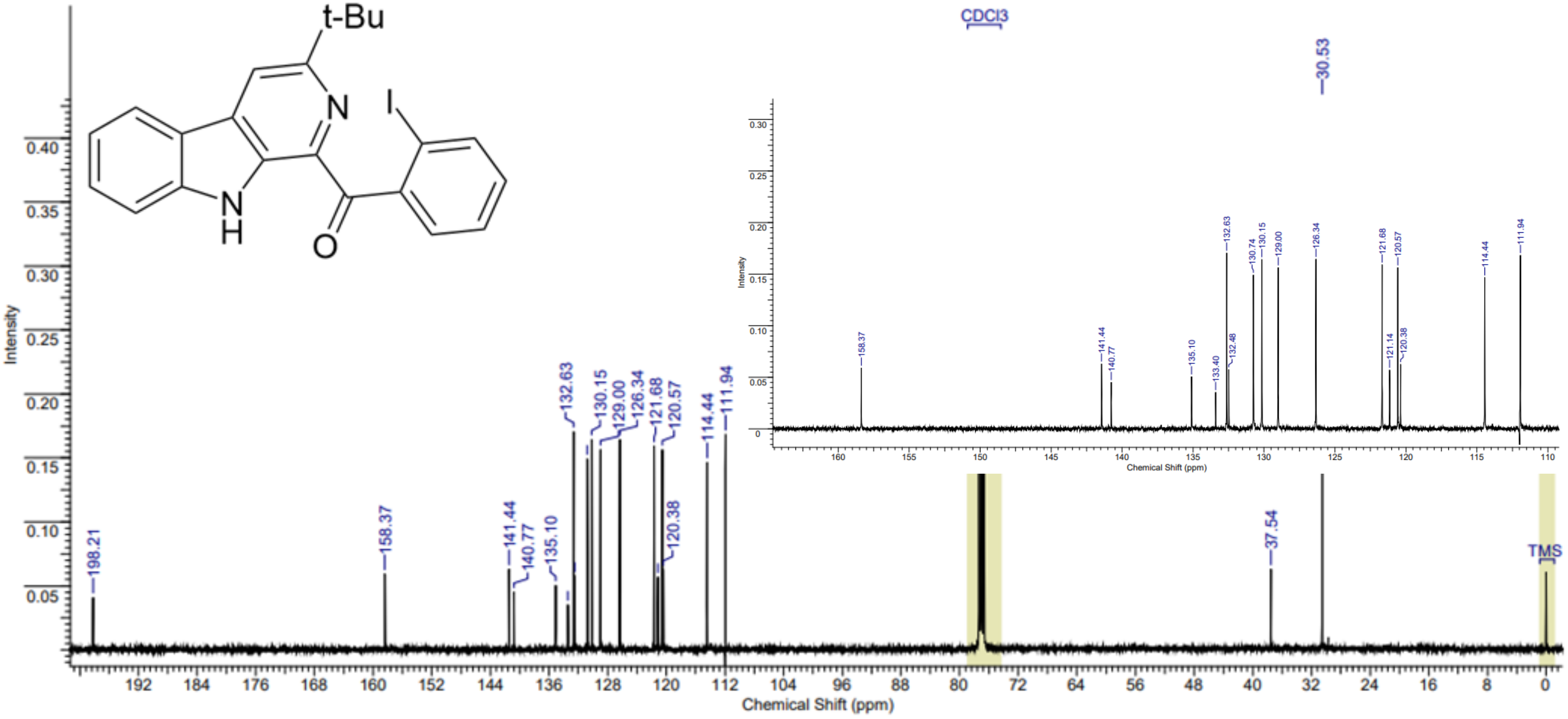

Figure S1. Spectra Data (contunuation)

^{1}H NMR spectra of 1-(2'-bromobenzoyl)-4-*tert*-butyl-β-carboline (13).

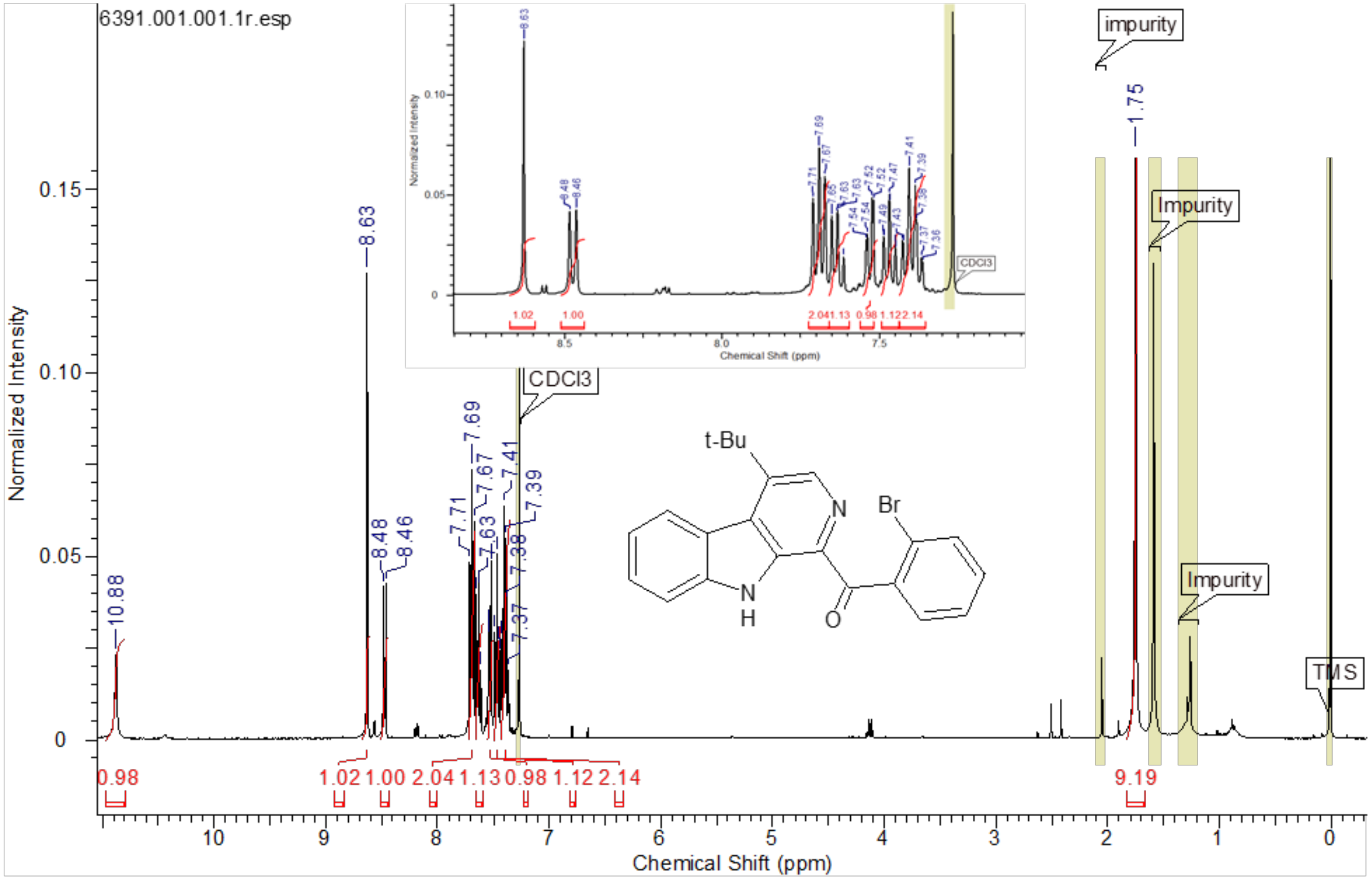

Figure S1. Spectra Data (contunuation)

^{13}C NMR spectra of 1-(2'-bromobenzoyl)-4-*tert*-butyl-β-carboline (13).

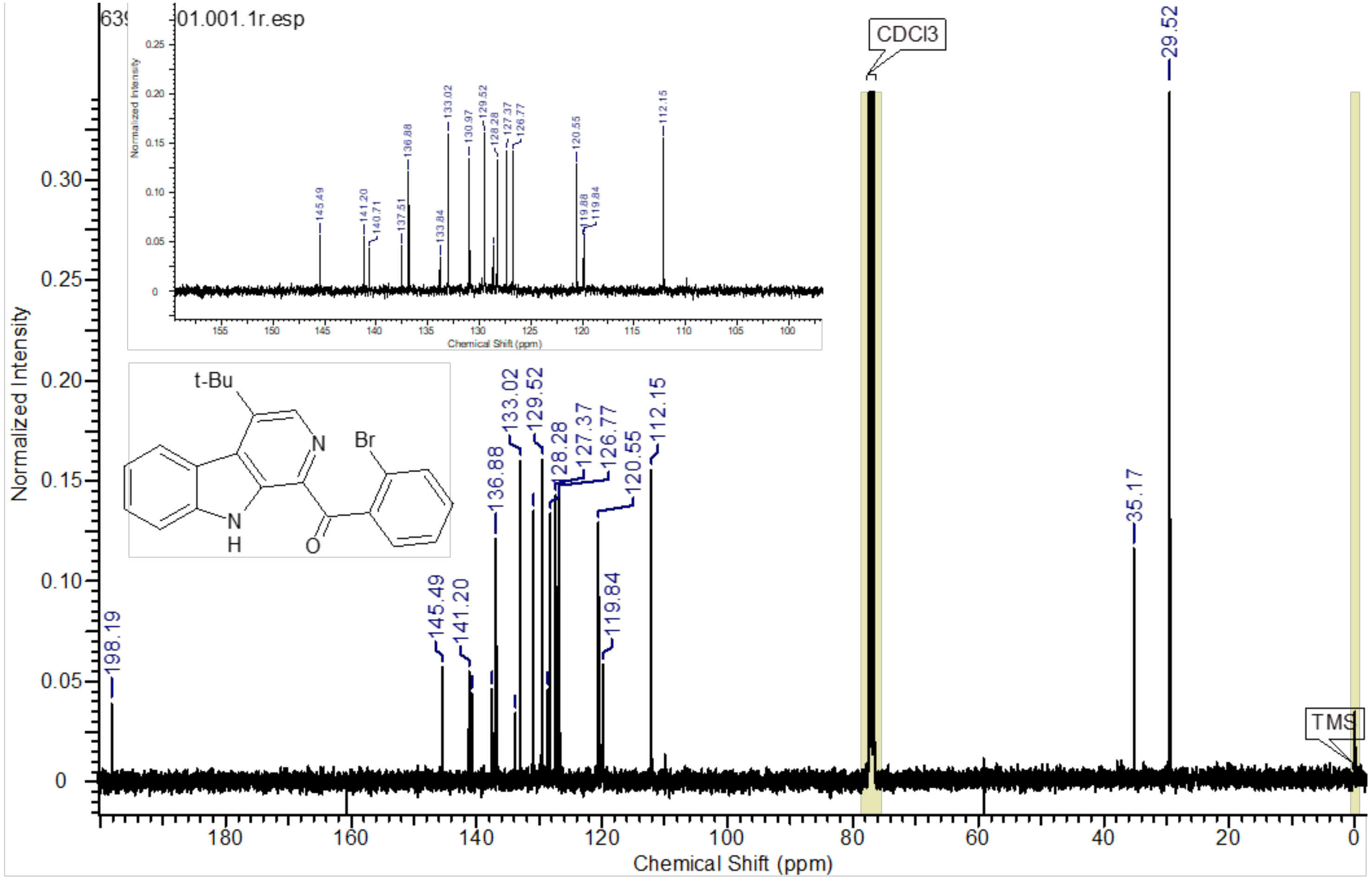

Figure S1. Spectra Data (contunuation)

^{1}H NMR spectra of 12,13-dihydro-6-*tert*-butyl-13-oxopyrido[1,2-a:3,4-b']diindol-5-ium (6-TB)

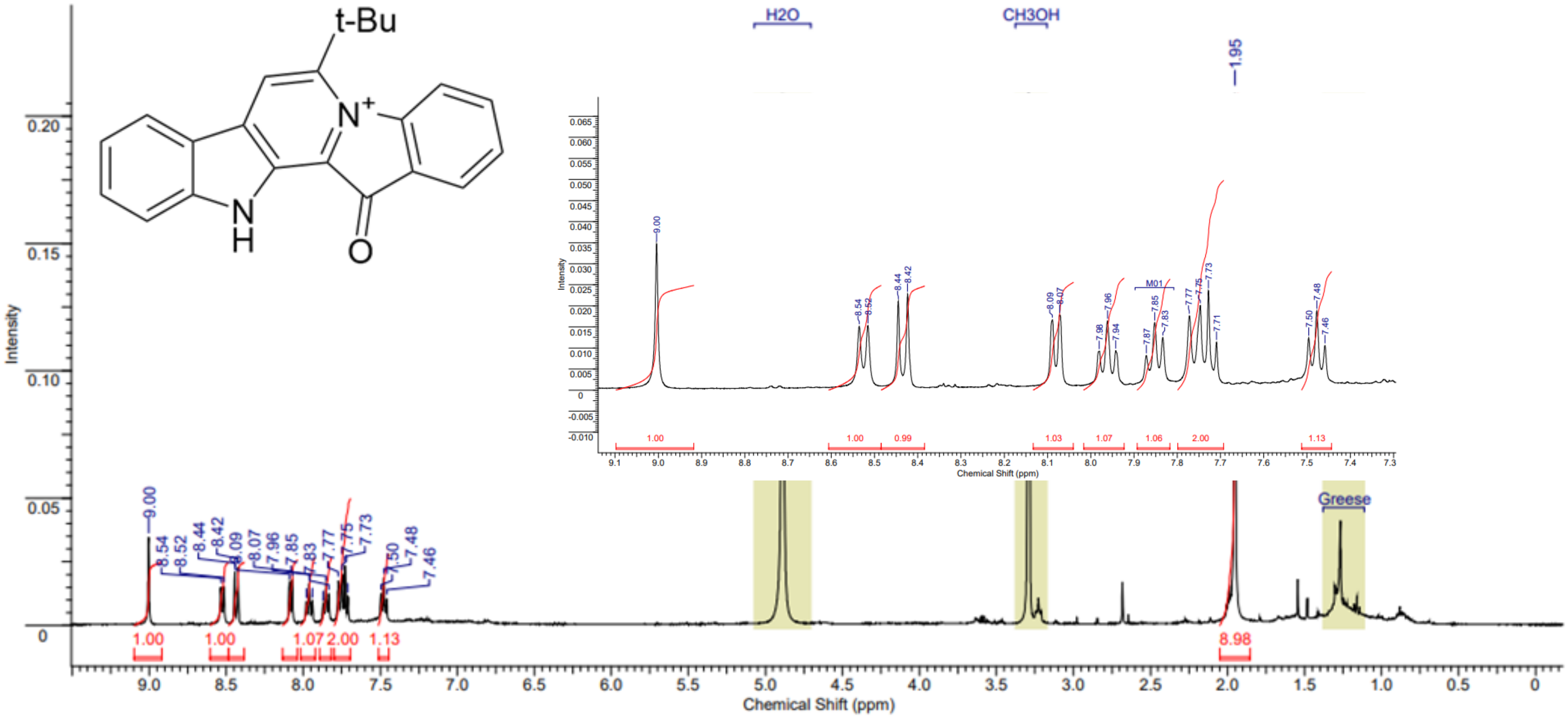

Figure S1. Spectra Data (contunuation)

^{13}C NMR spectra of 12,13-dihydro-6-*tert*-butyl-13-oxopyrido[1,2-a:3,4-b']diindol-5-ium (6-TB)

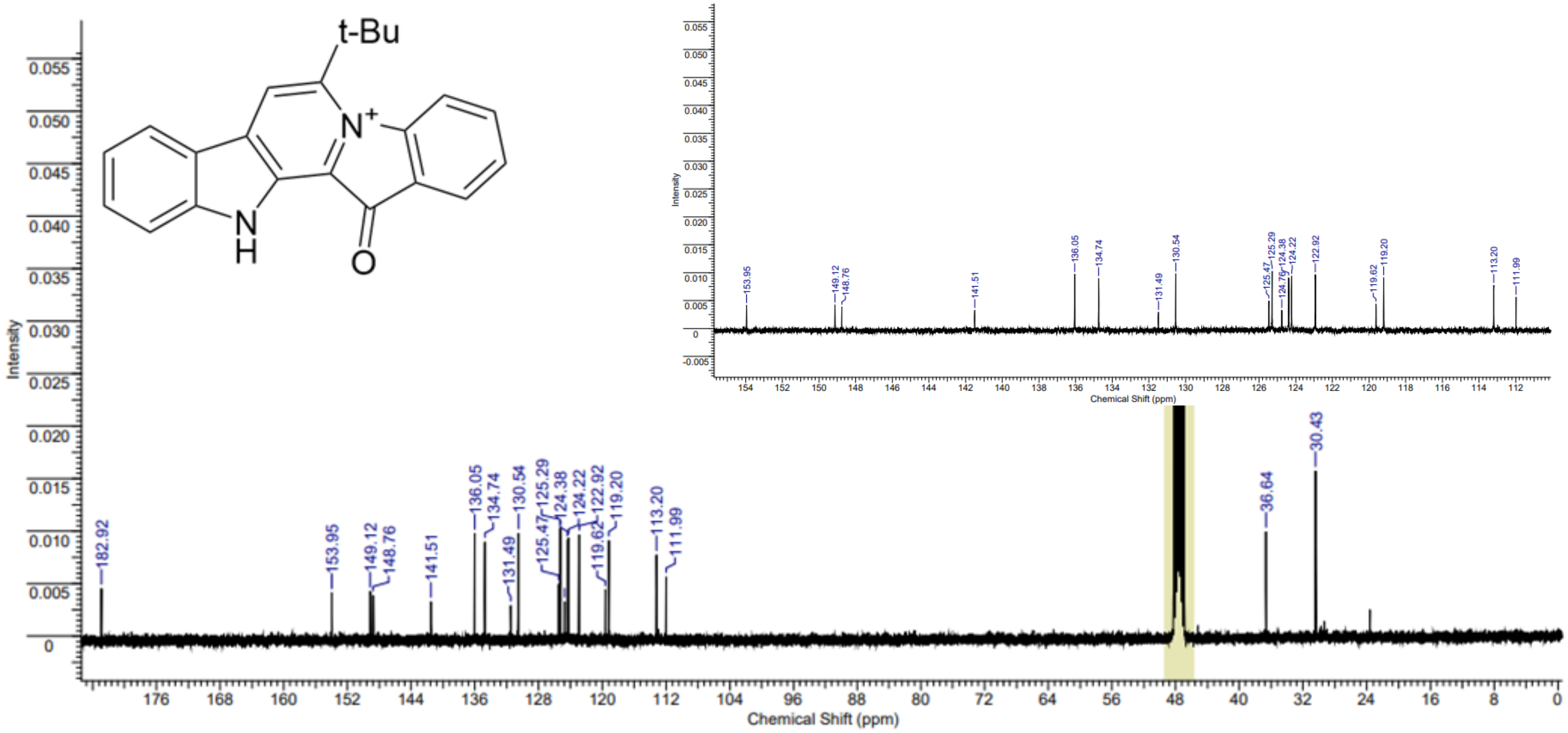

Figure S1. Spectra Data (contunuation)

^{1}H NMR spectra of 7-*tert*-butylfascaplysin (7-TB)

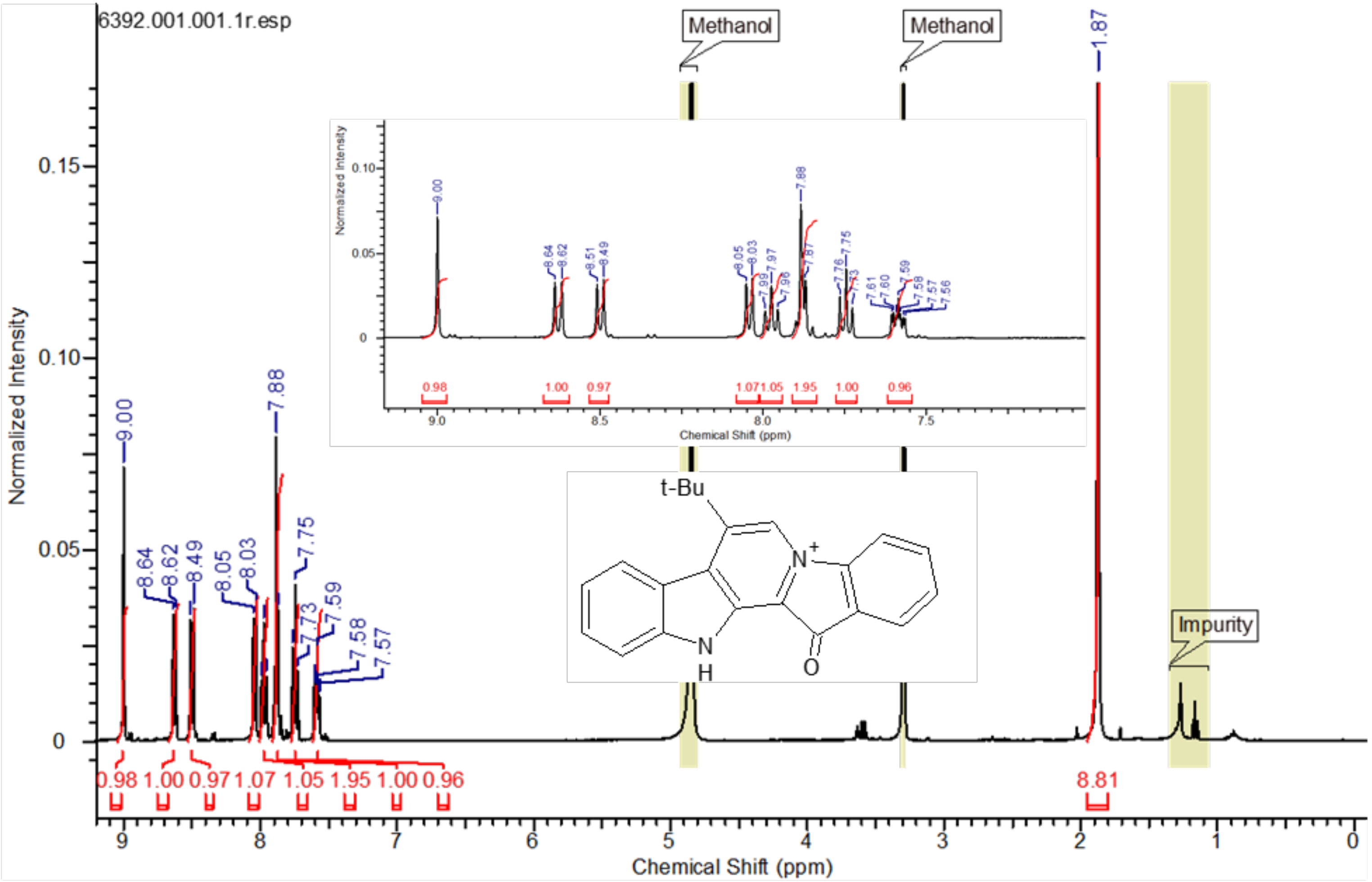

Figure S1. Spectra Data (contunuation)

^{13}C NMR spectra of 7-*tert*-butylfascaplysin (7-TB).

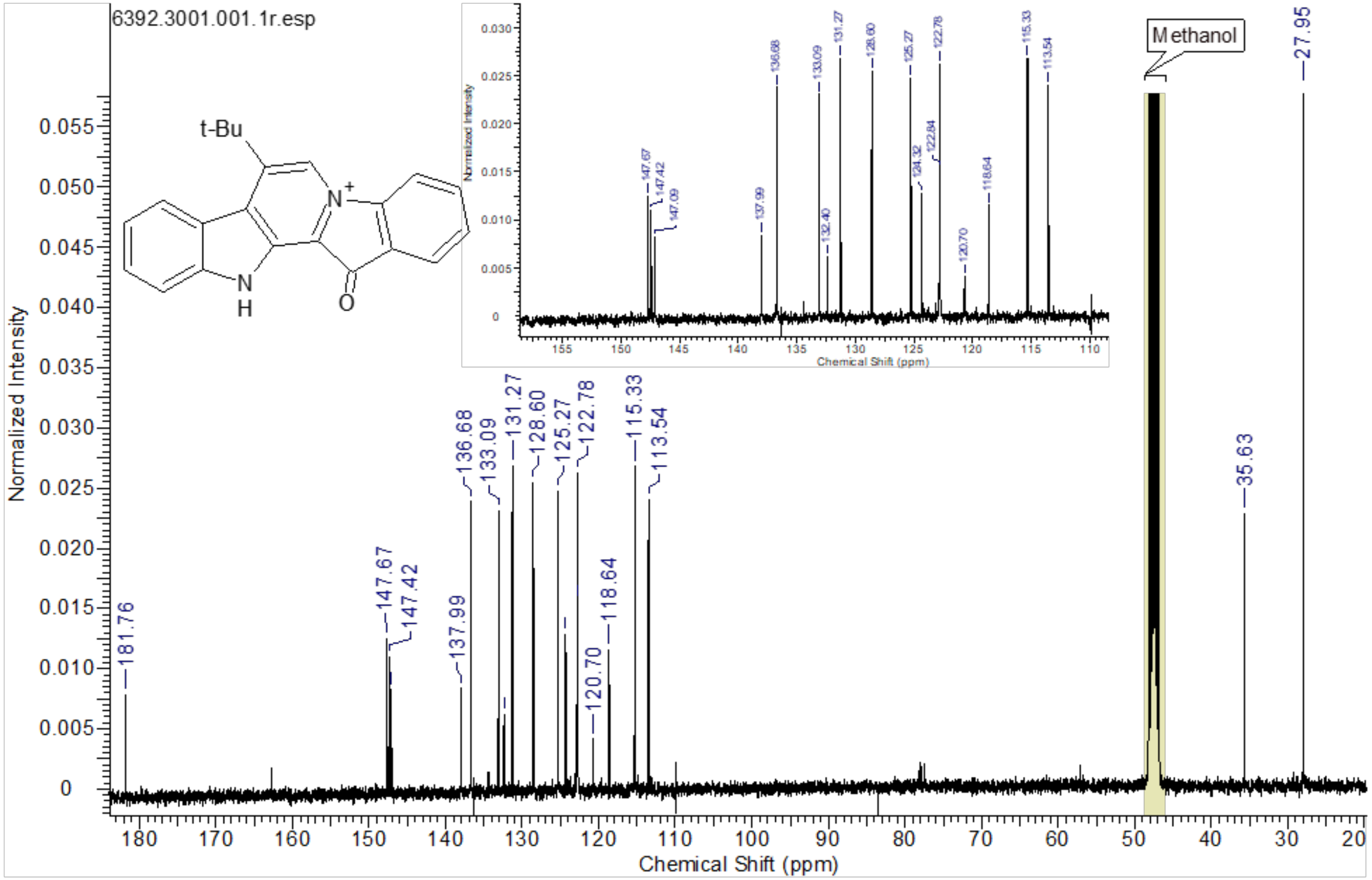

Table S1. Primary and secondary antibody used.

Antibodies	Clonality	Source	Cat.-No.	Dilution	Manufacturer
Rb	mAb	mouse	9309	1:1000	Cell Signaling
phospho-Rb (Ser807/811)	mAb	rabbit	8516	1:1000	Cell Signaling
anti-p21$^{Waf1/Cip1}$	mAb	rabbit	2947	1:1000	Cell Signaling
anti-α-Tubulin	mAb	mouse	T5168	1:5000	Sigma-Aldrich
anti-β-Actin-HRP	pAb	goat	sc-1616	1:10000	Santa Cruz
ATR	mAb	mouse	sc-515173	1:1000	Santa Cruz
phospho-ATR (Thr1989)	pAb	rabbit	58014	1:1000	Cell Signaling
ATM (2C1)	mAb	mouse	GTX70103	1:1000	GeneTEX
phospho-ATM (pS1981)	pAb	rabbit	GTX132146	1:1000	GeneTEX
Mre11 (12D7)	mAb	mouse	ab214	1:1000	abcam
CHK1 (2G1D5)	mAb	mouse	2360	1:1000	Cell Signaling
phospho-CHK1 (pS345) (133D3)	mAb	rabbit	2348	1:1000	Cell Signaling
CHK2	mAb	mouse	611570	1:1000	BD
phospho-CHK2 (Thr68)	pAb	rabbit	2661	1:1000	Cell Signaling
Ku70	mAb	mouse	NB100-1915	1:1000	Novus Biologicals
Ku80	pAb	rabbit	2753	1:1000	Cell Signaling
phospho-S139-H2AX	mAb	Mouse	05-636	1:500	Millipore
53BP1	pAb	rabbit	NB100-304	1:500	Novus Biologicals
anti-rabbit Alexa-fluor488		goat	A32731	1:600	Invitrogen
anti-mouse Alexa-fluor594		donkey	A32744	1:500	Invitrogen
anti-mouse IgG-HRP		sheep	NXA931	1:10000	GE Healthcare
anti-rabbit IgG-HRP		goat	7074	1:5000	Cell Signaling

Figure S2. Original images used for Figure 5A.

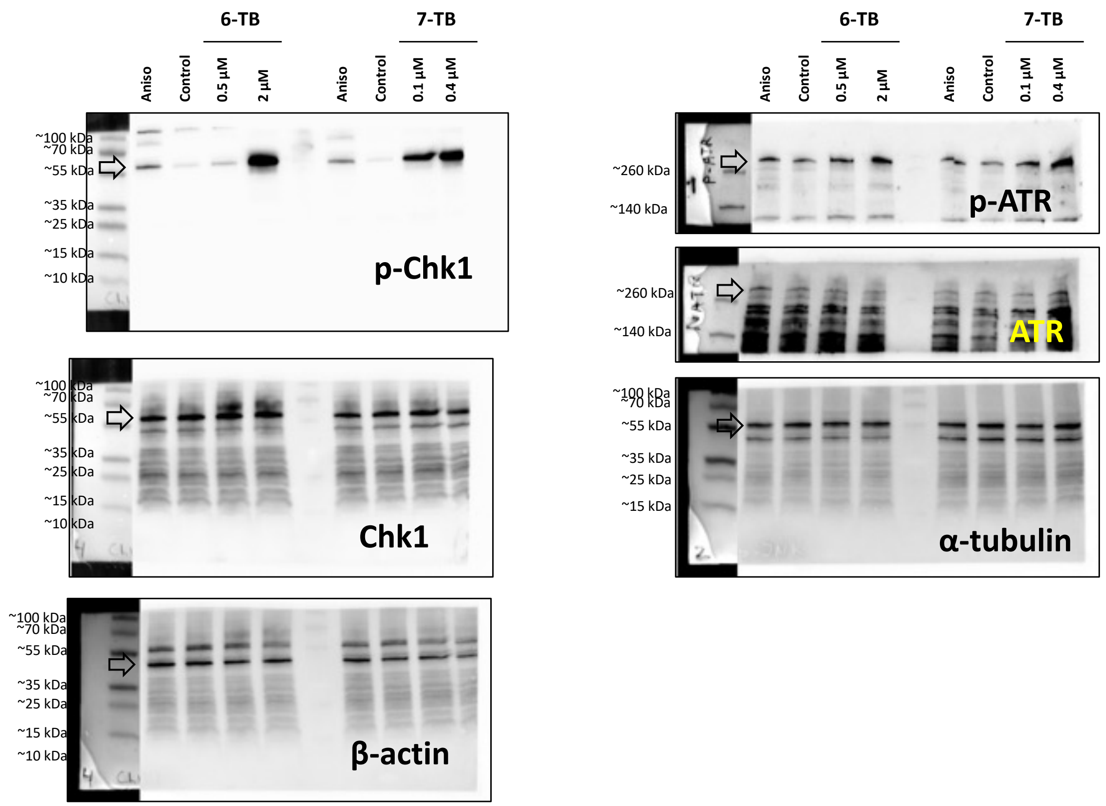

Figure S2. Original images used for Figure 5A (continuation).

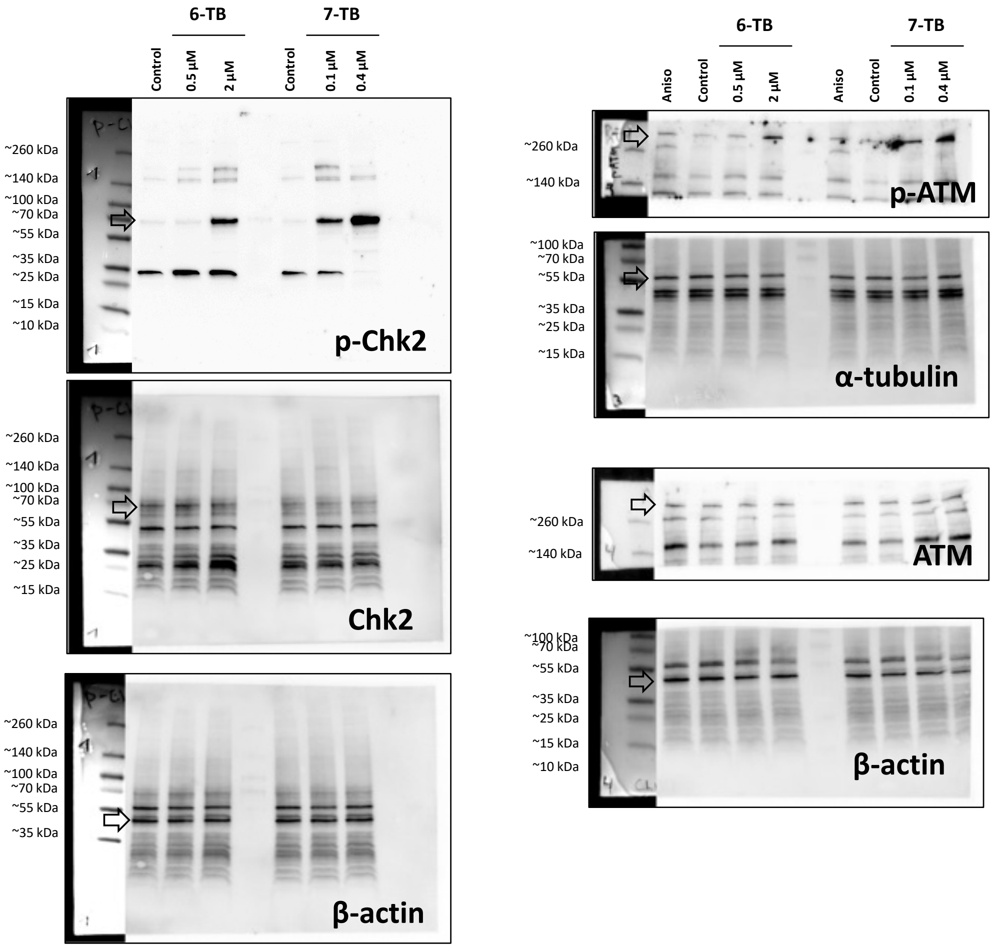

Figure S2. Original images used for Figure 5A (continuation).

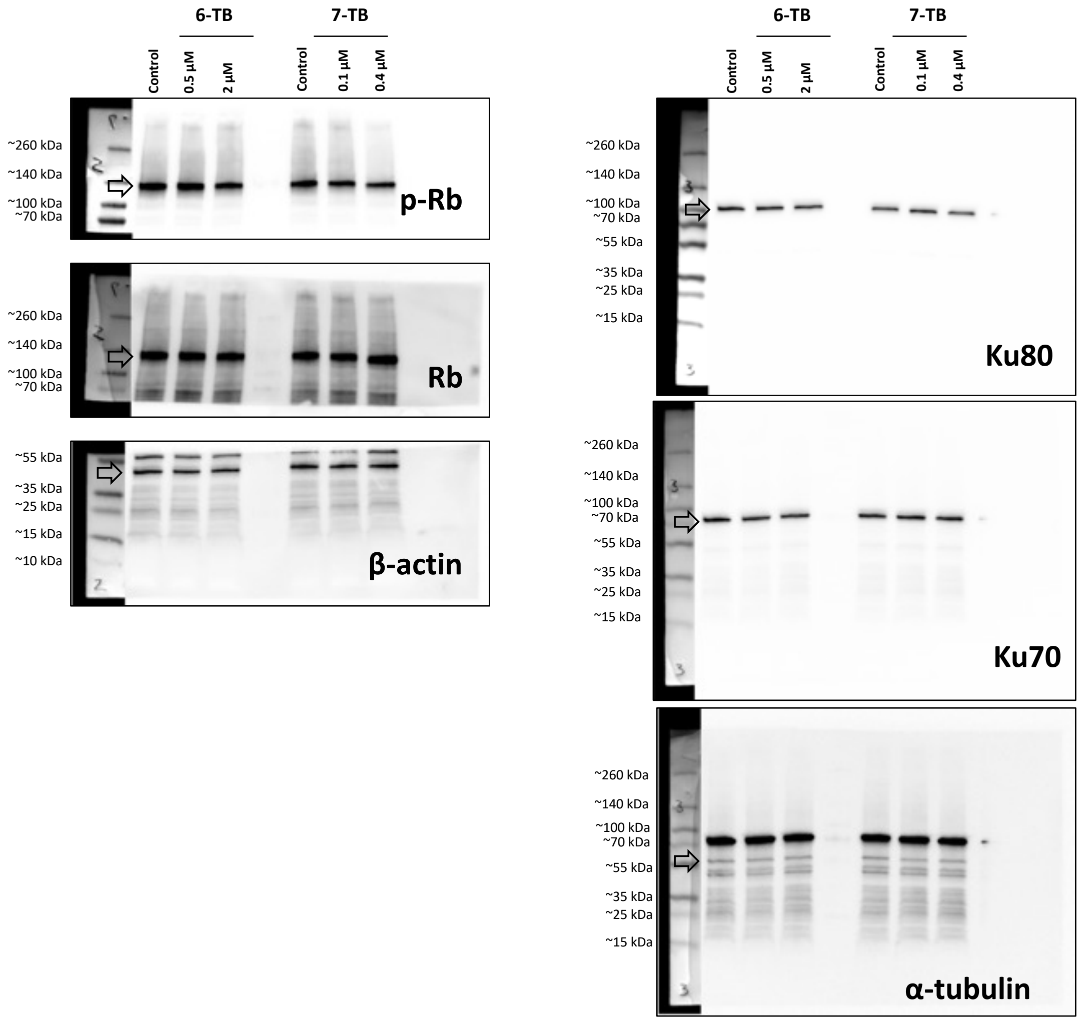

Figure S2. Original images used for Figure 5A (continuation).

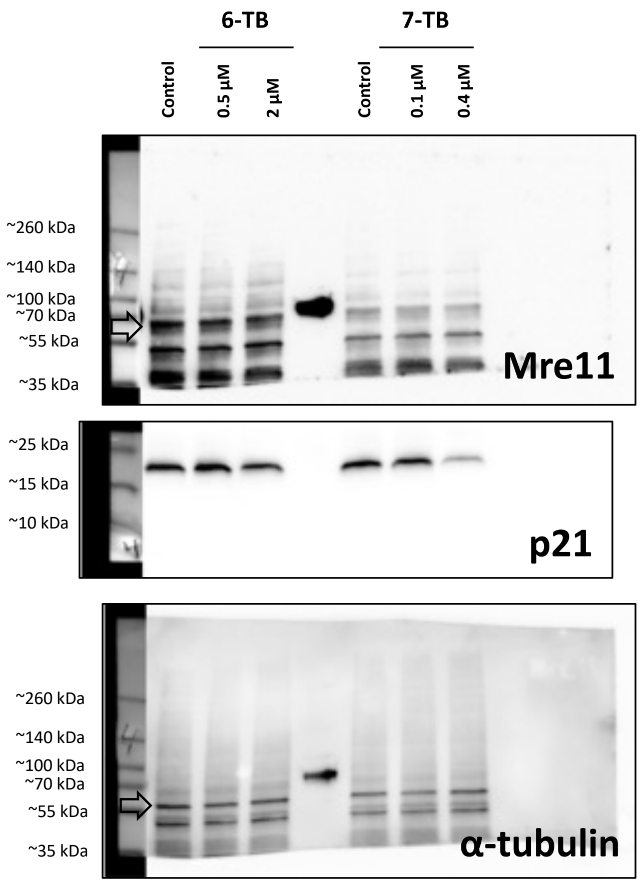

Supplement: Supplementary file 1 — Supplementary Information. [file 41598_2024_62358_MOESM1_ESM.pdf]
